# Multitarget Compounds Designed for Alzheimer, Parkinson, and Huntington Neurodegeneration Diseases

**DOI:** 10.3390/ph18060831

**Published:** 2025-06-01

**Authors:** Eleftheria-Emmanouela Katsoulaki, Dimitrios Dimopoulos, Dimitra Hadjipavlou-Litina

**Affiliations:** Laboratory of Pharmaceutical Chemistry, School of Pharmacy, Faculty of Health Sciences, Aristotle University of Thessaloniki, 54124 Thessaloniki, Greece; elefkats@pharm.auth.gr (E.-E.K.); ddimitrios@pharm.auth.gr (D.D.)

**Keywords:** Alzheimer’s disease, Parkinson’s disease, Huntington’s disease, neurodegenerative diseases, multitarget drugs, hybrid compounds

## Abstract

Multitarget drugs are molecules with the ability to act simultaneously on different targets at the same time, and they have been evaluated in the last decade as a powerful tool in the development of promising therapeutics for neurodegenerative diseases. This is very useful for multifactorial diseases such as Alzheimer’s, Parkinson’s, and Huntington’s diseases, a group of neurological disorders that induce neurodegeneration and neuroinflammation. Successful drug design for multifactorial diseases depends on an interdisciplinary and collaborative approach. The complexity of the above pathologies has clearly demonstrated that such single-target drugs are inadequate to achieve a successful therapeutic result. Furthermore, molecules hitting more than one biological target exhibit also a safer profile. In this review, we present a comprehensive knowledge of recent research on multitarget synthetic approaches to confront Alzheimer’s, Parkinson’s, and Huntington’s neurodegenerative diseases.

## 1. Introduction

### 1.1. Neurodegeneration and Multitarget-Directed Ligands

Neurodegenerative diseases, such as Alzheimer’s disease (AD), Parkinson’s disease (PD), and Huntington’s disease (HD), represent a class of chronic, progressive disorders characterized by the loss of neuronal structure and function. These conditions are associated with devastating clinical outcomes, including cognitive decline, motor dysfunction, and behavioral disturbances [1,2,3].

The pathophysiology of these disorders is multifaceted, involving oxidative stress, mitochondrial dysfunction, protein misfolding, and excitotoxicity. Traditional drug discovery approaches focusing on single molecular targets have often failed to halt or slow disease progression, highlighting the need for more integrative strategies [2,3,4].

In this context, multitarget-directed ligands (MTDLs) have emerged as a promising therapeutic concept. These compounds are rationally designed to interact with multiple pathological targets simultaneously, offering the potential to modulate the complex biochemical networks underlying neurodegeneration more effectively than single-target drugs [5,6,7,8,9].

This review aims to explore the latest advances of 2024 as well as some publications not reviewed from 2022 and 2023 in the design and application of MTDLs for AD, PD, and HD, emphasizing their pharmacological profiles, mechanisms of action, and the therapeutic rationale for their development.

### 1.2. Inclusion Criteria

To capture emerging trends, we prioritized peer-reviewed preclinical studies published in 2024 for AD, in 2023 and 2024 for PD, and all available recent studies for HD, given its limited literature. Many foundational MTDL candidates have already been covered extensively in earlier reviews; thus, this article emphasizes the most recent contributions to the field, aiming to highlight novel strategies and chemical entities that reflect current research priorities. From each selected article, we extracted and presented the most representative compounds, emphasizing their pharmacological strengths and, where applicable, discussing reported limitations (e.g., poor blood–brain barrier permeability). Each molecule was analyzed to the extent that the source article allowed, with particular attention to the therapeutic target combinations it aimed to address.

### 1.3. Multitarget-Directed Ligands

The present paradigm of “one drug, one target, one disease” is unlike to treat multifactorial neurodegenerative diseases such as AD, PD, and HD, offering only a palliative benefit. Complex diseases can be approached by administering multiple drugs that hit multiple targets (i.e., drug cocktail or fixed-dose combination), or using one drug to bind to MTDLs. Combination therapy has several drawbacks involving poor bioavailability and pharmacokinetic properties, drug–drug interactions, and resistance. On the other hand, MTDLs are engineered as a single molecule targeting various pathological entities and include numerous benefits: 1. Increased therapeutic effectiveness; 2. Lower toxicity; 3. Improved compliance and enhanced quality of life; 4. Avoid increased suppression or activation of a single pathway. Undoubtedly, the benefits come with corresponding costs: 1. Bigger molecule size leading to limited bioavailability; 2. Increased lipophilicity, causing high hepatic clearance; 3. Complexity in identifying appropriate targets [5,6,7,8,9].

**Figure 1:**
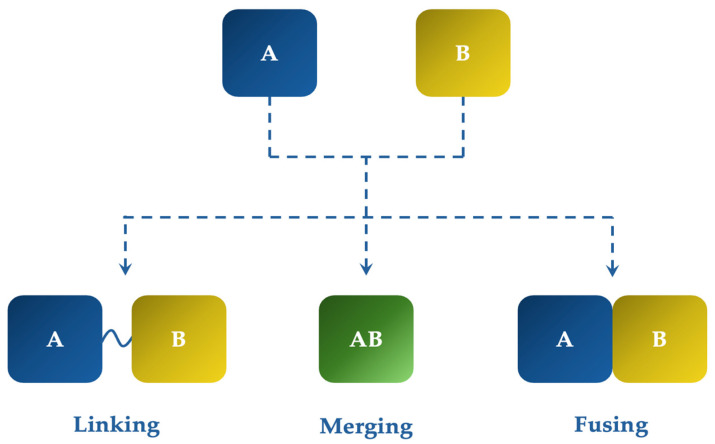
Different approaches to creating MTDLs.

MTDLs can arise from the linking, fusing, or merging method (Figure 1). The linking method involves the connection of two distinct pharmacophores “A” and “B” (with additive or synergistic effect) by using a linker. Conversely, the fusing method does not involve a linker to connect the two pharmacophores, hence lowering the molecular weight and favoring the blood–brain barrier (BBB) permeability. The merging method involves the integration of pharmacophores—key fragments—into a single molecule [5,6,7,8,9].

### 1.4. Alzheimer’s Disease

People suffering from AD were referred around 50 million in 2018, and it is estimated to triple by 2050, making it one of the most expensive and lethal diseases of this century [4,10]. AD progresses through three broad stages: preclinical, mild cognitive impairment, and dementia, marked by increasing cognitive decline [1]. Clinically, this progression is more precisely captured by the Global Deterioration Scale (GDS), which classifies seven stages from no impairment to severe dementia [11]. Pathologically, AD is staged using the ABC classification system, combining amyloid plaque distribution (Thal phases), neurofibrillary tangle spread (Braak stages), and neuritic plaque density (CERAD score) [12]. This dual clinical-pathological framework enhances understanding of disease progression and supports more accurate therapeutic evaluation. Increased risk factors for AD are unhealthy lifestyle behaviors such as lack of exercise, unhealthy diet, metabolic disorders including diabetes, cardiovascular disease, and stress [4,13]. Also, heritable factors can play a significant role [10,13,14].

AD is associated with various factors, including amyloid beta peptides (Aβ) aggregation, metal ion dyshomeostasis, and oxidative stress. The Cholinergic Hypothesis links reduced acetylcholine levels to cognitive decline, while the Amyloid Cascade Hypothesis focuses on Aβ aggregation and tau protein tangles. The Oxidative Stress Hypothesis highlights the role of reactive oxygen species in causing cellular damage, with therapeutic strategies targeting metal chelation to mitigate oxidative stress [1,13,14,15,16,17].

**Figure 2:**
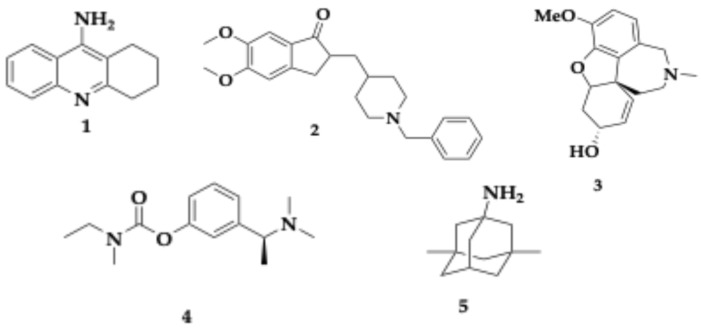
Compounds against AD.

Many biological systems are involved in the pathogenesis of AD. Currently, almost all the available drugs are palliative rather than curative, raising questions about the established strategy of “one drug, one target, one disease”. Treatments available now (Figure 2) include donepezil (molecule **2**), galantamine (molecule **3**), rivastigmine (molecule **4**), and memantine (molecule **5**). The first three are acetylcholinesterase inhibitors (AChEI), which conserve the acetylcholine (Ach) levels and are approved for mild-to-moderate AD. Memantine, used to treat moderate-to-severe AD, is an *N*-methyl-D-aspartate (NMDA) antagonist. Another AChEI is tacrine (THA, molecule **1**), which was withdrawn due to its hepatotoxicity [1,13,18,19].

Although most approved therapies for AD remain symptomatic, recent advances are challenging the conventional single-target drug model by introducing agents that address the underlying pathophysiology of the disease. Particularly, sodium oligomannate (GV-971), approved in China, represents a multi-target strategy that modulates gut microbiota, suppresses neuroinflammation, and influences metabolic pathways [20,21,22]. In parallel, monoclonal antibodies such as lecanemab (BAN2401) and donanemab (LY3002813) have received conditional approval in the United States and other regions based on their ability to reduce Aβ burden and slightly slow cognitive decline [23,24]. These developments underscore the increasing recognition that a successful therapeutic approach to multifactorial disorders like AD must extend beyond single-target strategies, highlighting the clinical relevance and necessity of MTDLs.

### 1.5. Parkinson’s Disease

PD absolute number of cases has increased, due to the heightened life expectancy, and as cases continue to arise, some scientists describe it as a pandemic [2,25,26]. PD is a neurodegenerative disorder affecting primarily the motor system, causing resting tremors, bradykinesia, postural instability, and gait impairment [27,28]. Lewy bodies, protein aggregates in substantia nigra neurons, can cause PD [27,29]. Synaptic damage and mitochondrial dysfunction are initial steps in the development of PD. Dopaminergic neurons require a high amount of energy to work properly, so the mitochondrial normal bioenergetic function is significant for their survival. Changes in mitochondrial structure and function are associated with higher levels of reactive oxygen species, abnormal calcium levels inside cells, and decreased production of adenosine triphosphate (ATP) by mitochondria [29]. Similarly to AD, PD can have a hereditary basis [27,29,30].

**Figure 3:**
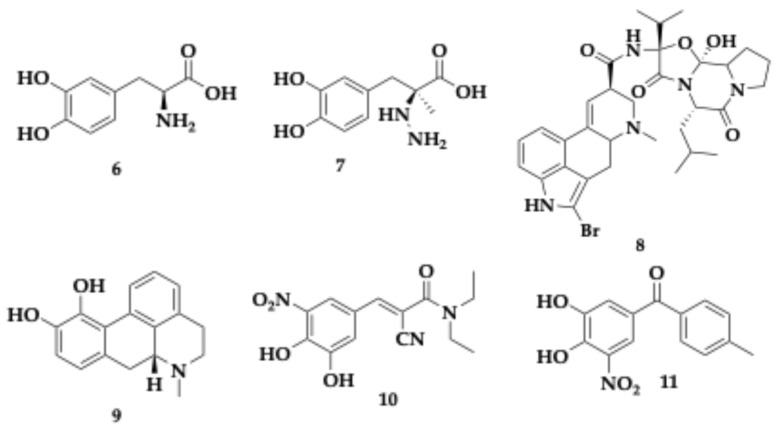
Compounds against PD.

Multiple defects can be attributed to the complexity of neurodegeneration in PD and AD. Pathological features observed in both conditions are the progressive decline in neurotransmission and the formation of deleterious protein aggregates. Primary enzyme targets to regulate the neurotransmitter levels used as therapeutic agents are: monoamine oxidases (MAOs) and catechol-O-methyltransferase (COMT). Symptomatic improvement is the only benefit of using these therapeutic approaches, as they are unable to modify the course of the disease, and their efficacy wears off, parallel with motor complications. Administration of catecholamines as carbidopa and L-3,4-Dihydroxyphenylalanine (L-DOPA) is used to restore dopaminergic tone. Agonists for dopaminergic receptors are also available (Figure 3) and include, among others, bromocriptine (molecule **8**), apomorphine (molecule **9**), cabergoline, pramipexole, piribedil, and ropinirole. To enhance the central availability of dopamine, L-DOPA (molecule **6**) is commonly coadministered with carbidopa (molecule **7**), which prevents premature metabolism of L-DOPA before it crosses the blood–brain barrier. In addition, COMT inhibitors such as entacapone (molecule **10**) and tolcapone (molecule **11**) are used to prolong dopaminergic tone by blocking peripheral and central COMT-mediated degradation of L-DOPA [1,17,28,29,31].

### 1.6. Huntington’s Disease

Globally, HD affects 2.7–17.2 out of every 100,000 people, depending on regional factors [3]. HD is a neurodegenerative and inherited condition affecting motor, and cognitive functionality and causing psychiatric conditions. These symptoms progressively lead to the loss of voluntary motor control-increased involuntary movements (Huntington’s chorea), dementia, and premature lethality [3,32,33]. Aspiration pneumonia and cardiovascular complications are the most common causes of death [3,33]. The mechanisms of neurodegeneration in HD include NMDA receptor-mediated excitotoxicity, dopaminergic dysfunction, mitochondrial dysfunction, oxidative stress, impaired autophagy, abnormal protein aggregation, disrupted gene transcription, and loss of trophic support, among others [3,34].

**Figure 4:**
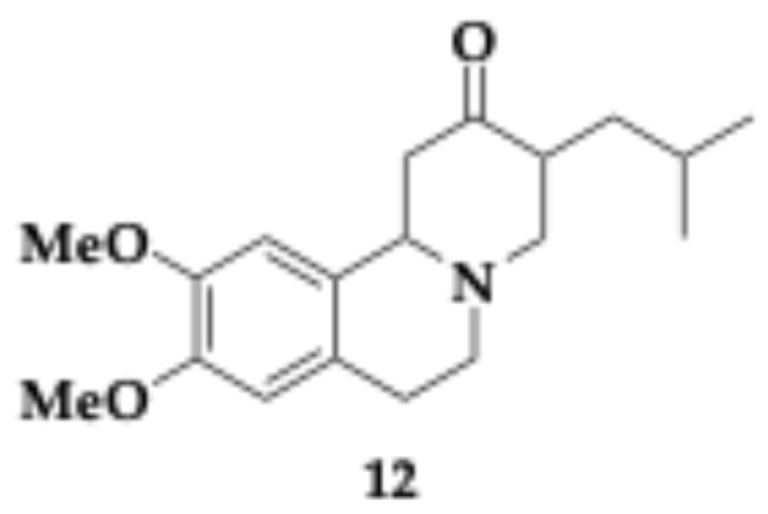
Compound against HD.

So far, clinical trials have not succeeded in finding disease-modifying treatments for HD, while the existing therapies mainly focus on symptom management. Current treatments address motor, cognitive, and psychiatric symptoms, aiming to enhance the quality of life for patients. Tetrabenazine (Figure 4, molecule **12**) is used to decrease movement disorders. Furthermore, antidepressants, antipsychotics, and tranquilizers are administered as a medication [3,35].

## 2. Multitarget Compounds Against AD

### 2.1. Inhibition of Cholinesterases and Aβ Aggregation

Bon et al. [36] designed and evaluated nine rivastigmine-indole MTDLs. The two coupled moieties are able to inhibit both acetylcholinesterase (AchE) and butyrylcholinesterase (BuChE), Aβ_42_ aggregation, biometal chelation, and present antioxidant activity. Indole scaffold has an acknowledged neuroprotective role, while endogenous molecules containing it (e.g., melatonin, serotonin) seem to be decreased in AD patients. Human AChE and BuChE have a catalytic active site (CAS), a peripheral anionic site (PAS), and the same catalytic triad. The molecular docking of these MTDLs supported the inhibitory effect, as all molecules were able to establish interactions with important residues on both enzymes.

The lead compounds **13** and **14** (Table 1) demonstrated higher AChE inhibition than the parent rivastigmine drug (**13**; Inhibitory Concentration 50 (IC_50_) = 10.9 μM, **14**; IC_50_ = 26.8 μM). By increasing the linker, the authors observed an enhancement in the AChE inhibition ability. The BuChE inhibition is not related to the linker’s length. The free radical scavenging activity of **13** and **14** appeared to be stronger than vitamin C (**13**; Effective Concentration 50 (EC_50_) = 14.5 μM, **14**; EC_50_ = 20.7 μM); however, the *para*-substituted analogs are more active. The hydroxyl group is vital, as molecules not containing it present reduced activity. Τhe most effective **14** molecule inhibits the Aβ_42_ aggregation by 55.5%, and antimyloidogenic activity is also susceptible to the position of the hydroxyl group in the indole moiety. Higher inhibitory activity was observed by **13** and **14** at Cu-induced Aβ_42_ aggregation, as these molecules are able to chelate copper. Both **13** and **14** presented neuroprotective effects and increased SK-N-SH human neuroblastoma cell line (SH-SY5Y) viability. In silico evaluation has also been conducted, suggesting that the hybrids have the potential for oral bioavailability [36].

Mishra et al. [37] executed several in silico, in vitro, and in vivo experiments and identified molecule **15** as a potential MTDL against AD. Compound **15** is a benzothiazole-piperazine derivative capable of inhibiting AChE and Aβ_1–42_ aggregation competently. The acetamide linker provided enough flexibility for the benzothiazole moiety to interact with AChE’s CAS, while PAS engages with the piperazine pharmacophore. Molecule **15** inhibited AChE with an IC_50_ value of 0.42 μM, whereas standard drug donepezil displayed an IC_50_ value of 0.049 μM. Selective inhibition of AChE is crucial for developing safe anti-AD molecules, due to the possibility of cholinergic distress caused by BuChE’s inhibition, making molecule **15** a safe candidate (BuChE; IC_50_ > 100 μM). Regarding inhibition of self and Cu-mediated Aβ_1–42_ aggregation, compound **15** displayed a stronger inhibitory effect than the reference compound curcumin (**15**; 80.70%, curcumin; 50.23%). In vitro evaluation exhibited neuroprotective potential against H_2_O_2_ and okadaic acid toxicity in SH-SY5Y and Neuroblastoma-2A cell lines (Neuro2A). Furthermore, **15** seemed to enhance spatial memory and learning in a dementia model induced by scopolamine.

Nasr et al. [38] evaluated thiazole-piperazine hybrids as anti-AD agents. The novel MTDLs were tested for their ability to inhibit AChE, BuChE, Aβ aggregation and showed metal chelating activity. The most potent compound **16** was able to effectively inhibit cholinesterases (AChE; IC_50_ = 0.151 μM, BuChE; IC_50_ = 0.135 μM). A significant inhibitory effect was observed regarding Aβ_1–42_ aggregation (73.53%). The docking study revealed the interaction of **16** with key fragments in CAS and PAS of AChE. Cytotoxicity assessment on SH-SY5Y and pheochromocytoma 12 cell lines (PC12) indicated no harmful effects. Furthermore, compound **16** effectively reduced Aβ-induced toxicity and protected SH-SY5Y cells at a concentration of 10 μM. Similarly, molecule **17** presented remarkable activity against the aforementioned targets (AChE; IC_50_ = 0.499 μM, BuChE; IC_50_ = 0.103 μM, Aβ aggregation inhibition; 79.42%), demonstrating simultaneously safety and effectiveness. Both molecules were predicted to cross the BBB in silico, and further pharmacokinetic studies suggested druglikeness following Lipinski’s rule of five.

Singh et al. [39] designed and thoroughly evaluated ferulic acid-piperazine derivatives against major targets of AD’s pathology. The most advantageous compound **18** inhibited cholinesterases (AChE; IC_50_ = 0.59 μM, BuChE; IC_50_ = 5.02 μM), Aβ_1–42_ aggregation, and presented antioxidant and metal chelating properties. Furthermore, it was able to inhibit the nucleotide-binding domain, leucine-rich-containing family, and pyrin domain-containing-3 (NLRP30) inflammasome, which are involved in AD pathogenesis by promoting Aβ aggregation. Kinetic studies revealed a reversible inhibitory effect on AChE and BuChE by the benzyl derivative **18,** and the propidium iodide assay suggested efficient interaction with PAS. The antioxidant nature of the compound was confirmed, with an estimated IC_50_ value of 5.88 μM. Molecule **18** is able to chelate iron ions, inhibit self- and metal-induced Aβ aggregation, and show no cytotoxic effects on PC12 for concentrations up to 30 μM. Parallel artificial membrane permeability assay (PAMPA) established appropriate BBB permeability, and **18** managed to reduce cellular and mitochondrial reactive oxygen species (ROS). In vivo testing indicated no toxicity for doses up to 550 mg/kg. Additionally, improvement in spatial and learning abilities in animal scopolamine-induced amnesia models was observed when **18** were administered at 5 mg/kg. Further ex vivo experiments demonstrated the multitherapeutic abilities of **18**, as it reduced malondialdehyde quantities and elevated superoxide dismutase and catalase levels.

Manzoor et al. [40] discovered sixteen potential candidates for dual AChE and Aβ aggregation inhibitory activity. Among the tested compounds, the most potent AChE inhibitors were **19** and **20**, with estimated IC50 values of 1.29 and 1.72 μM, respectively. None of the designed molecules violated Lipinski’s rule, also they presented encouraging BBB permeability and oral bioavailability. Regarding the Structure-Activity Relationship (SAR), the electron-withdrawing group at position number 3 of **19** and the electron-donating group at position number 4 of **20** are significant for the activity against AChE. In addition, at a dose-dependent rate, both molecules inhibited the aggregation of Aβ_42_ (**19**; IC_50_ = 4.39 μM, **20**; IC_50_ = 1.42 μM) and displayed superior neuroprotective activity on SH-SY5Y cells compared to donepezil. The antioxidant ability of the compounds was assessed and revealed a reduction in nitric oxide and malondialdehyde quantities, and elevated glutathione, superoxide dismutase, and catalase levels. PAMPA suggested that both **19** and **20** are able to cross the BBB, and the immunohistochemical evaluation highlighted the inhibition of Aβ aggregation. The authors suggest that their findings can lead to further optimization of future AD therapeutics.

Zeng et al. [41] studied twenty-seven L-tryptophan derivatives. A promising candidate compound **21** exhibited potent, mixed, and selective inhibition of BuChE (IC_50_ = 0.44 μM). The free radical scavenging potential of **21** was determined to be effective in aqueous solutions. In addition to the BuChE inhibition, hydroxyl and peroxide radical scavenging by **21** was observed. Self-induced Aβ_42_ aggregation was inhibited by 52.50%, and cytocompatibility was observed for concentrations up to 25 μΜ at PC12 and AML12 cell lines. The PAMPA suggested good BBB crossing, and in silico testing verified the significant oral bioavailability and safety regarding the cardiac toxicity.

Zaafar et al. [42] continued their research, designing twenty-eight 5-substituted-2-anilino-1,3,4-oxadiazole hybrids, and the leading hit managed to surpass the rivastigmine inhibitory activity. Specifically, compound **22** presented significant inhibition against AChE and BuChE (AChE; IC_50_ = 46.9 nM, BuChE; IC_50_ = 3.5 nM). In the PbAc-induced animal model of AD, the administration of **22** presented antioxidant potency, reducing malondialdehyde levels while increasing glutathione. Decreased levels of AChE and BuChE were also observed, while self-induced Aβ aggregation was inhibited. In addition, the histopathological and immunohistochemical evaluation suggested the neuroprotective and anti-apoptotic nature of **22**, considering the ameliorated histological damage followed by the reduction in caspase-3 and vascular endothelial growth factor (VEGF). In silico tools predicted efficient BBB crossing, molecular docking, and cellular dynamics studies revealed good stability and binding affinity of the molecule.

Wang et al. [43] reported another novel melatonin-hydroxyquinoline hybrids capable of chelating biometals and reducing oxidative stress triggered by hydrogen peroxide. Utilizing the neuroprotective effect of melatonin, molecules **23** and **24** appeared to have better scavenging ability than the parent compound, the position of the linker on the hydroxyquinoline moiety significantly impacts the activity, decreasing it when placed at position number 7. Regarding the inhibition of Aβ_1–42_ aggregation, the activity is enhanced using as connection mode the amide and hydroxyl group as a substituent on the indole ring at position 5. Compound **24** has the greatest inhibition rate at 63.24%. The chelation ratio of molecules **23** and **24** is 2:1, **23** significantly inhibits Cu-induced Aβ_1–42_ aggregation, exceeding the reference compound clioquinol. Cytotoxicity assay was conducted on SH-SY5Y and murine microglial cell line (BV2) cell lines, and no toxic effect was reported at 5 μM and 20 μM concentrations, respectively, of **23** and **24**. PAMPA shows that most of the hybrids can cross the BBB. Poor permeability was observed only by compounds with -OH substituent as a result of the increased hydrophilicity.

### 2.2. Inhibition of GSK-3β

Qiu et al. [44] designed and studied twenty-nine harmine derivatives to effectively inhibit glycogen synthase kinase-3β (GSK-3β) and dual-specificity tyrosine phosphorylation-regulated kinase 1A (DYRK1A). In AD patients, GSK-3β is overexpressed in the brain, leading to tau hyperphosphorylation, while DYRK1A’s excessive activation influences the stability and regulation of tau protein. Hyperphosphorylated tau accumulates and promotes neurofibrillary tangles (NFTs) formation, one of the main pathological features of AD.

The lead compound **25** (Table 2) exhibited great inhibitory effects on GSK-3β and DYRK1A (GSK-3β; IC_50_ = 66 nM, DYRK1A; IC_50_ = 111 nM), far superior to the parent compound harmine. **25** interacts with the ATP binding pocket of these two enzymes. The combination of the methoxy group at position 7 with the introduction of a fluorine atom substituent leads to potent activity. Additionally, the aliphatic chain length and amide group at position 1 of the linker are vital for sustaining functionality. The PAMPA demonstrated possible BBB permeability, and optimal gastrointestinal absorption was predicted using the SwissADME platform. No cytotoxic effect was spotted in SHSY-5Y and human liver 7702 cell lines (HL-7702), until a 20 μM concentration of **25**. Inhibition of tau phosphorylation tested on the okadaic acid-induced SHSY-5Y cell model was observed, whereas **25** managed to reduce NFTs in a concentration-dependent way [44].

Wu et al. [45] tested novel tetrahydroacridin hybrids with sulfur-inserted linkers as potential anti-AD MTDLs. The optimal molecule **26**, designed as a hybrid of tacrine and pyrimidone, inhibited AChE and GSK-3β (AChE; IC_50_ = 0.047 μM, GSK-3β; IC_50_ = 0.930 μM). Kinetic study and molecular modeling studies confirmed that **26** managed to occupy the CAS and PAS catalytic sites of AChE simultaneously. The cystamine group linker provided enough flexibility to ensure the concurrent inhibition of both AChE’s catalytic sites and retained activity regarding the inhibition of GSK-3β. Assays conducted on SH-SY5Y cells showed no toxicity at concentrations up to 25μM. Compound **26** was tested for possible hepatotoxicity, as a tacrine hybrid, on Hepatocellular Gep 2 cells (HepG2). Although no notable hepatotoxicity was observed at concentrations up to 20 μM, the authors suggested further optimization for improved outcomes. Finally, poor BBB permeability was predicted. Molecule **26** seems to violate Lipinski’s rule, regarding molecular weight.

Abdo et al. [46] designed novel quinoline-2-one derivatives and reported interesting findings regarding the GSK-3β inhibition. Among several promising molecules, compound **27** exhibited potent inhibitory activity against GSK-3β (IC_50_ = 6.68 nM) and managed to effectively reduce tau aggregation. The SAR demonstrated that the superior inhibition justified by the balance of hydrophilic tail and kinetic studies revealed the competitive inhibition. Molecular docking simulation showed that **27** could occupy the ATP binding site by utilizing the quinoline-2-one ring and establishing hydrogen bonds. No important cytotoxic effects were observed at Telomerase-Immortalized Human Liver Epithelial Cells 2 (THLE2) and SH-SY5Y cells, while in silico testing predicted no violation of Lipinski’s rule and good brain penetration. **27** enhanced the cognitive ability when administered in a scopolamine-induced mouse model. It is worth emphasizing that **27** demonstrated low selectivity against GSK-3β, indicating interaction with enzymes such as Cyclin-Dependent Kinase 2 (CDK2) and other kinases.

### 2.3. Inhibition of HDAC

Santini et al. [47] reported another series of potential disease-modifying MTDLs for AD by combining histone deacetylase (HDAC) and GSK-3β inhibition. HDAC enzymes are involved in neuronal viability and cognitive function by modulating gene expression and the function of non-histone proteins, such as tau. Herein, inhibition of different HDAC isoforms, mainly HDAC2 and HDAC6, is associated with neuroprotective effects. Three distinct structural moieties are required for molecules with anti-HDAC activity, including a zinc-binding group, a catabolite activator protein (CAP) group, a large aromatic surface, and a linker. Due to the highly similar structure between isoforms, selective inhibition can be proved challenging.

The most promising compound **28** (Table 3) performs as a non-ATP-competitive GSK-3β inhibitor and therefore provides fewer off-target effects and decreased toxicity. Based on the balanced inhibitory profile of molecule **28** (GSK-3β; IC_50_ = 0.142 μM, HDAC2; IC_50_ = 0.030 μM, HDAC6; IC_50_ = 0.045 μM) further studies conducted. In neuronal SH-SY5Y cells, major toxicity was observed only at concentrations exceeding 25 μM, and Western blotting analysis revealed significant activity of compound **28** against the targeted enzymes. Furthermore, in a dose-related manner, restriction of CuSO_4_-mediated tau phosphorylation and neuroprotective effects were observed during in vitro testing. Another major component of AD pathogenesis is regulated by the immunomodulatory activity of compound **28**, in relation to neuroinflammation and microglia activation. In silico screening predicted no violation of Lipinski’s rule, and PAMPA demonstrated good BBB permeability. Finally, authors unveiled ongoing trials to optimize molecule pharmacokinetic and pharmacodynamic parameters [47]

Another potential HDAC6 inhibitor for AD treatment, compound **29**, was further evaluated by Liu et al. [48] on Aβ/Cu-induced rat models. The results indicated reduced levels of Aβ, tau, and hyperphoshorylated tau; therefore, inhibition of neurofibrillary tangle formation was exhibited. Alteration of mRNA expression related to neuronal apoptosis was observed (e.g., downregulation of Caspase-3 and Bax mRNA). Additionally, regulation of oxidative stress and attenuation of the neuroinflammatory response were discovered in rats treated with **29**.

Diniz et al. [49] explored the activity of **30**, a novel HDAC6 inhibitor, as a promising therapeutic approach for AD due to its multifaceted effects on neuroinflammation and synaptic health. The authors conducted extensive tests in both in vitro and in vivo models to assess its efficacy. In cultured primary neural hippocampal cells, **30** demonstrated no cytotoxic effects while effectively reducing HDAC activity and increasing histone acetylation, crucial for gene expression regulation. In an animal model of AD induced by Aβ oligomers, **30** modulated astrocyte reactivity, decreased the expression of pro-inflammatory markers such as TNF-α and IFN-γ, and promoted a shift from a neurotoxic A1 astrocyte phenotype to a neuroprotective A2 phenotype. Additionally, **30** enhanced synaptogenic properties of astrocytes which was evident from the increase in synapse formation and preservation of synaptic proteins.

### 2.4. Inhibition of Cholinesterases and Histamine Antagonism

Pérez et al. [50] designed and tested pitolisant-sulfonylurea derivatives to inhibit AChE and demonstrate histamine H3 receptor (H_3_R) antagonism, as a novel MTDL approach. H_3_R antagonism is correlated to histamine and ACh release, leading to the treatment of cholinergic deficits causing cognitive impairments. Lead compound **31** (Table 4) was characterized by the authors as an optimal starting point for further investigation and development, since it exhibited the highest AChE inhibition (AChE; IC_50_ = 7.65 μM, H_3_R; IC_50_ = 0.13 μM). Furthermore, PAMPA was performed, and Absorption, Distribution, Metabolism, and Excretion (ADME) properties were predicted. Molecule **31** suggested satisfactory BBB permeability, did not violate Lipinski’s rule of five, and enhanced cognitive function in the scopolamine-induced AD mouse model.

Michalska et al. [51] provided a promising basis for future structure optimization by designing 4-oxypiperidine ethers as anti-AD agents. Authors targeted with the synthesized compounds the inhibition of AChE, BuChE, and the antagonism/inverse agonism of histamine H_3_R. One of the most promising molecules, **32**, presented significant interaction with the aforementioned enzymes (H_3_R; inhibition constant (K_i_) = 12.5 nΜ, AChE; IC_50_ = 1.537 μM, BuChE; IC_50_ = 1.353 μM). The decreased flexibility due to the rigid naphthalene ring of **32** provided improved interaction with the binding site.

Chen et al. [52] managed to explore the first scutellarein 7 L-amino acid carbamate-4′-cycloalkylamine propyl ether conjugates as novel MTDLs against AD. The most favorable compound **33** presented excellent AChE inhibitory activity and H_3_R antagonism superior to clobenpropit (AChE; IC_50_ = 9.73 μM, H_3_R; IC_50_ = 1.09 nM). Furthermore, **33** can interact with both catalytic sites of AChE (PAS and CAS) and inhibit self- and Cu- induced Aβ aggregation by 94.58% and 88.63%, respectively. Disaggregation of Aβ fibrils was also observed, while Aβ_25-35_ induced tau hyperphosphorylation was decreased. Antioxidant potency and neuroprotective effects monitored at PC12 cell lines against H_2_O_2_ injury. Additional in vitro testing suggested good BBB penetration at human Cerebral Microvascular Endothelial Cells (hCMEC/D3) and human Peptide Transporter 1 Madin-Darby Canine Kidney cells (hPepT1-MDCK). In the AD model, compound **33** enhanced spatial memory and cognitive function when administered to rats, and in vivo assessment validated the favorable pharmacokinetic profile.

### 2.5. Compounds Against Neuroinflammation

Carrieri et al. [53] developed and investigated ten enantiomeric pairs, utilizing a pyrrolidine scaffold, as neuroprotective MTDLs for potential treatment of AD. The authors managed to inhibit AChE, BuChE, and fatty-acid amide hydrolase 1 (FAAH) enzymes with the tested *N*-substituted pyrrolidine derivatives. The endocannabinoid system includes the FAAH enzyme, which degrades endogenous cannabinoids, which are involved in decreasing neuroinflammation and regulating memory and locomotion. In AD patients, high levels of FAAH are obtained in the brain, therefore making its inhibition a reasonable target.

Most promising compounds (R)-**34** (AChE; IC_50_ = 0.215 μM) and (S)-**35** (BuChE; IC_50_ = 0.155 μM) showed sufficient inhibitory activity on AChE and BuChE, respectively (Table 5). Stereochemistry had a considerable impact on the activity profile, revealed by the estimated four times higher IC_50_ of (S)-**35** on BuChE. Moreover, (S)-**35** IC_50_ value is equivalent to 0.94 μM regarding FAAH inhibition. Regarding the antioxidant activity, the majority of the (R) isomer compounds managed to reduce H_2_O_2_-induced oxidation in HepG2. Molecule (R)-**34** overcame the reference molecule quercetin, a strong antioxidant, with an IC_50_ value of 0.40 μM. No cytotoxic effects were detected for concentrations up to 100 μM on HepG2 and SH-SY5Y cell lines. Although all compounds were anticipated to exhibit strong binding to albumin, only (S)-**35** was identified as having good BBB permeability, as it is suggested by the PAMPA [53].

Fotopoulos et al. [54] designed and evaluated novel multifunctional hybrids to target neuroinflammation in AD and PD. Although inflammation-related enzymes are consistently present in certain regions of the brain, in AD patients, elevated translation of lipoxygenase (LOX) and cyclooxygenase-2 (COX2) leads to Aβ production. Therefore, they present a promising therapeutic target in combination with lipid peroxidation inhibition and anti-inflammatory activity.

The authors developed cinnamic amide hybrids with γ-aminobutyric acid, L-glutamic acid, and glycine and tested them for their activity. Regarding lipid peroxidation, the most potent compound **36**, a glycinate hybrid, demonstrated a 99% inhibition rate. In vitro evaluation was conducted on soybean isoenzyme LOX-1, and the most active compound was **37** (IC_50_ = 8.5 μM). The most effective COX-2 inhibitor was found to be a glycinate hybrid **38** (IC_50_ = 5 μM). To achieve simultaneous dual inhibition, a compound like **39** presents a viable option. Additionally, compound **37** shows no violation of Lipinski’s rule of five, but unfavorable BBB permeability is estimated theoretically; in this context, the structural characteristics of the amino acid may be demonstrated to confer benefits [54].

Asghar et al. [55] tested tryptamine derivatives as a multifactorial approach against AD. Different SAR approaches revealed multiple potent inhibitors, and the most promising **40** showed superior activity compared to tryptamine and donepezil (AChE; IC_50_ = 0.70 μM, MAO-B; IC_50_ = 43.21 μM). Furthermore, it managed to inhibit COX-2 by 75.16%. Compound **40** is a meta-di-nitro benzoyl derivative, able to interact with both PAS and CAS of AChE. Previous beliefs regarding the nitro group were disproven, as the electron-withdrawing group significantly influences the potency, and a change to the position of the group (para- or meta-) seems to decrease the inhibitory potential. The authors suggest further optimization and evaluation of the promising **40** compounds.

Waiker et al. [56] designed eighteen diaryl triazine hybrids to discover effective anti-AD agents. One of the top candidates, compound **41** was tested thoroughly and was determined as a potent inhibitor regarding AChE and Beta-site Amyloid precursor protein Cleaving Enzyme-1 (BACE-1) (AChE; IC_50_ = 0.486 μM, BACE-1; IC_50_ = 0.542 μM). Molecule **41** consists of electron-donating groups at the 3 and 5 positions of the phenyl ring, exhibits selectivity against AChE (BuChE; IC_50_ > 10 μM), and interacts with both CAS and PAS. Permeability of the BBB is predicted to be excellent by the PAMPA, and no neurotoxicity was observed at SH-SY5Y cells at the highest concentration (80 μM). Compound **41** was able to inhibit up to 81.1% of the Aβ_1–42_ aggregation and exhibited neuroprotective properties. In silico studies predicted no violation of Lipinski’s rule and acceptable oral bioavailability. In animal models, **41** proved to be safe for doses up to 1000 mg/kg, and no alterations in multiple tissues were observed. Scopolamine-induced rat models exhibited enhanced memory when treated with **41** (2.5–10.0 mg/kg). The hippocampal tissue of the examined mice underwent further analysis, and ex vivo studies demonstrated a recovery of cholinergic-related cognitive function, showing equipotent activity to donepezil. Immunohistochemical examination revealed a remarkable decline of BACE-1 and Aβ. Western blot verified previous findings, as reduced levels of tau and Amyloid Precursor Protein (APP) were observed. Finally, compound **41** reintegrated the Aβ-induced phenotype in the Drosophila model.

Authors reported another synthesized compound, **42**, that managed to inhibit DYRK1A with an estimated IC_50_ value of 2 μM. DYRK1A is a protein kinase overexpressed in AD patients and is located in reactive astrocytes and microglia, which are involved in neuroinflammation [56].

Moftah et al. [57] evaluated a series of twenty quinazolinone-based hybrids as potential anti-AChE compounds. One of the leading compounds **43** presented positive AChE inhibition (23.8%), anti-inflammatory and antioxidant activity. The Morris water maze test exhibited significant enhancement of cognitive function when treated with compound **43**. The neuroinflammation is considered an important aspect of AD’s pathophysiology. Thus, important inflammatory cytokines evaluated upon **43** treatment and results revealed inhibition of TNF-a, nuclear factor kappa-light-chain-enhancer of activated B cells (NF-κΒ), Interleukin-1 beta (IL-1β), and Interleukin-6 (IL-6) by up to approximately 25%. Histopathological examination exhibited equipotent neuroprotective effects of **43** with donepezil and underlined that substitution with an electron-withdrawing group significantly impacts the activity against AChE. Molecular docking simulations displayed that the most active derivatives have the necessary structural features for binding, explaining their selectivity through docking scores and binding patterns.

Zhang et al. [58] developed eighteen bakuchiol–coumarin MTDLs, and the most advantageous compound **44** presented efficient inhibition against AChE (IC_50_ = 34.78 μM). A molecular docking study revealed an interaction with both PAS and CAS of AChE. The results suggest that substitutions at the 3, 6, and 7 positions of the coumarin moiety can positively impact AChE activity. One advantage of compound **44** is its anti-inflammatory properties, since a concentration of 5 μΜ effectively inhibited TNF-α (53.89%) and IL-6 (86.53%) production. Authors encourage additional optimization, as in silico testing suggested a violation of Lipinski’s rule and poor BBB permeability.

Xia et al. [59] evaluated tryptanthrin hybrids as potential anti-AD agents. The most promising compound **45** presented significant reversible AChE inhibition (IC_50_ = 12.17 nM) and selectivity over BuChE, while molecular docking studies demonstrated that it was able to occupy both CAS and PAS of AChE. Regarding the SAR, open alkyl amine and a decreased number of alkyl groups enhanced the inhibitory activity. Furthermore, **45** presented remarkable anti-Aβ_1–42_ aggregation activity, compared to donepezil and curcumin. No cytotoxicity against BV2 and PC12 cell lines was observed for multiple concentrations. Compound **45** reduced inflammatory responses in LPS-induced BV2 cells by inhibiting multiple targets (nitric oxide (NO); IC_50_ = 2.13 μM, IL-1β; IC_50_ = 2.21 μM, TNF-α; IC_50_ = 2.47 μM). Additionally, demonstrated neuroprotective effects, copper chelating activity, and favorable BBB permeability. Compound **45** exhibited good tolerability at doses up to 101.56 mg/kg in mice and proved to enhance spatial cognitive function in scopolamine-induced in vivo models. The anti-inflammatory activity of **45** was confirmed by the observation of healthy neurons in multiple hippocampal regions.

### 2.6. Inhibition of Cholinesterases and MAOs

Huang et al. [60] designed and evaluated novel tacrine-selegiline hybrids to inhibit cholinesterases (AChE and BuChE) and monoaminoxidases (MAO-A and MAO-B). Dual inhibition of MAOs is preferred since MAO-A regulates important neurotransmitters in the brain as epinephrine, norepinephrine, and serotonin. MAO-B is overexpressed in the brains of AD patients and contributes to oxidative stress, as it generates enormous amounts of free radicals.

The most promising compound **46** (Table 6) showed balanced inhibitory activity (AChE; IC_50_ = 1.57 μM, BuChE; IC_50_ = 0.43 μM, MAO-A; IC_50_ = 2.3 μM, MAO-B; IC_50_ = 4.75 μM). Furthermore, **46** can occupy both PAS and CAS of AChE. Regarding the SAR, several compounds substituted with different groups at position 7 of the tacrine moiety were tested. Molecules containing benzyl ether appeared to have better inhibition against AChE. Concerning the MAO activity, compounds containing benzyl ether are preferable for MAO-A inhibition and vice versa regarding phenol ether. Sufficient permeability of the BBB is estimated for molecule **46** using the PAMPA, and further pharmacokinetic studies revealed acceptable properties. Compound **46** exceeded in vitro cytotoxicity, and during in vivo evaluation, doses up to 2500 mg/kg were tolerated. In the scopolamine–mouse model, molecule **46** managed to improve memory impairment in a dose-dependent manner [60].

Ayoup et al. [61] evaluated 1,2,4-oxadiazole derivatives for potential activity against AChE, BuChE, MAO-A, MAO-B, and oxidation, resulting in a positive outcome. The compound **47** contains a 3-benzyl substituent at the oxadiazole ring and is a potent, selective AChE inhibitor (IC_50_ = 0.023 μM) with equipotent to quercetin antioxidant activity (IC_50_ = 536.83 μM). Among the tested molecules, **48** exhibited significant inhibition against MAO-A (IC_50_ = 47.25 μM). Almost all compounds exhibited a safe profile, as suggested by the anti-hemolytic evaluation. The authors selected as the leading compound **49** due to its physicochemical properties.

Jalil et al. [62] examined quinoline-based sulfonamide hybrids as agents against AD and PD. Among the most significant synthesized compounds, **50** presented excellent inhibitory activity against the targeted enzymes (AChE; IC_50_ = 2.65 μM, BuChE; IC_50_ = 1.16 μM, MAO-A; IC_50_ = 1.34 μM, MAO-B; IC_50_ = 0.47 μM). In silico evaluation predicted no violation of Lipinski’s rule, favorable gastrointestinal absorption, and BBB penetration.

Sarfraz et al. [63] utilized in silico tools to design and evaluate chromen-2-one-based piperidine hybrids against AChE, BuChE, MAO-A, and MAO-B. The most promising compound **51** presented docking results better than the parent molecule, and favorable drug-likeness was predicted.

Finally, Fayyaz et al. [64] investigated in silico 3-phenylcoumarin derivatives as potential anti-AD agents and reported compound **52** as a promising candidate against cholinesterases and monoaminoxidases.

### 2.7. Compounds Against Secretases

Verma et al. [65] designed and investigated the biological properties of quinazoline derivatives as potential MTDLs against AChE and BACE-1. Among the 21 tested molecules, 3 demonstrated potent AChE inhibitory activity, and the most significant was compound **53** (AChE; IC_50_ = 0.193 μM, BuChE; IC_50_ > 10 μM, BACE-1; IC_50_ = 0.254 μM), and can be found in Table 7. In vitro data implied that the unsubstituted phenyl moiety, in combination with the benzyl piperazine substituent at the fourth position, provides enough flexibility and leads to superior BACE-1 inhibitory activity. The aromatic ring system is believed to provide satisfactory lipophilicity, and PAMPA confirmed the BBB permeability. Compound **53** remarkably displaced propidium iodide from the PAS region of AChE, and its anti-Aβ aggregation properties were validated experimentally. In more detail, self- and AChE-induced Aβ aggregation was inhibited by 29.18–48.72% and 60.38–69.00%, respectively. Non-neurotoxic effects were estimated for a concentration of up to 80 μM of **53** in SH-SY5Y cell lines. In vivo studies, utilizing the Y-maze and Morris water maze tests, demonstrated that compound **53** significantly enhanced learning and cognitive function in a dose-dependent manner. Ex vivo and biochemical analyses revealed that **53** markedly inhibited AChE activity and elevated ACh levels in the hippocampus. Moreover, oxidative stress markers were reduced, while levels of superoxide dismutase, glutathione, and catalase were notably increased following **53** treatment. The compound’s multitarget potential was confirmed by Western blot analysis, which showed a reduction in the expression of APP, Tau, and BACE-1. Immunohistochemistry further corroborated these findings, indicating decreased Aβ and BACE-1 expression in hippocampal tissues. Pharmacokinetic studies suggested that **53** possesses good oral absorption and brain permeability. The authors emphasize the significance of compound **53** as an anti-AD agent and suggest further research to explore its potential.

Tan et al. [66] investigated the role of thirty-seven cinnamamide/ester triazole hybrids as neuroprotective agents against AD. By exploiting the antioxidant and anti-inflammatory role of cinnamic acid, authors managed to effectively prevent Aβ production and to promote nonamyloidogenic pathways.

The linking method was applied to create the novel MTDLs. Beneficial was proved to be the benzylethylamine linker, related to enough flexibility, and enhanced the anti-AChE activity. The lead candidate compound **54** managed to inhibit copper-induced Aβ toxicity (EC_50_ = 1.39 μM), showing more potent activity than the reference drugs donepezil and memantine. Benzene ring considerably improves activity when substituted at positions 2 and 4, making the 4-trifluoromethyl compound **54** the most potent. Due to the significantly higher IC_50_ values for AChE inhibition observed in the tested compounds compared to donepezil, their activity is likely not driven by direct AChE inhibition. In vivo evaluation of compound **54** revealed no cytotoxicity for doses up to 25 mg/kg, and the bioavailability is predicted to be 44.0% for oral administration and 63.3% for intraperitoneal administration. Based on these data and the good brain permeability, further studies were conducted on molecule **54**. Among the most important findings, compound **54** successfully improved spatial cognitive ability in Aβ_1–42_ injured mice at different doses and presented higher activity than donepezil. On the contrary, Aβ aggregation and neuronal deterioration were limited [66].

Long et al. [67] designed and evaluated twenty capsaicin-tacrine hybrids as potential anti-AD therapeutics by inhibiting AChE, BuChE, and BACE-1. The hybrids emerged by linking the two scaffolds with a flexible aliphatic chain, and the most favorable compound **55** exhibited balanced activity (AChE; IC_50_ = 69.8 nM, BuChE; IC_50_ = 68.0 nM, BACE-1; IC_50_ = 3.6 μM). The long linker applied to **55** allowed the mixed-type inhibition of cholinesterases. The PAMPA revealed sufficient BBB permeation. Cytotoxicity testing on PC12 and BV2 cells suggested further investigation. Additional in vivo studies showed tolerance for doses up to 2500 mg/kg. No hepatotoxic effects were observed. The beneficial effects of **55** were confirmed when administered to mice, as it enhanced memory and limited latency and errors.

Bajad et al. [68] developed novel hybrids by bearing an *N*-aryl piperazine moiety to chalcone. The lead compound **56** was found to be a potent AChE and BuChE inhibitor (AChE; IC_50_ = 14.84 μM, BuChE; IC_50_ = 41.39 μM). Furthermore, at a concentration of 10 μM, it managed to inhibit BACE-1 (46.60%). The structure of compound **56** significantly influences its activity, with the substitution of a halogen at position 4 and the incorporation of benzylpiperazine proving to be the most effective configuration among those evaluated in the SAR study. Molecule **56** effectively inhibited self- and AChE-induced Aβ_1–42_ aggregation, and PAMPA revealed the ability to cross the BBB. In silico evaluation predicted no hepatotoxicity and compliance with Lipinski’s rule of five. In the scopolamine-induced mice model, a major enhancement of memory and cognitive abilities was observed when administered at doses of 50 mg/kg. A biochemical ex vivo analysis revealed increased levels of catalase and a reduction in malondialdehyde, underlying the antioxidant properties of **56**.

Verma et al. [69] further optimized quinazoline scaffolds and evaluated the novel MTDLs. The lead compound **57** presented inhibitory activity against AChE and BACE-1 (AChE; IC_50_ = 0.283 μM, BACE-1; IC_50_ = 0.231 μM). Authors introduced substituted piperazines at position four of quinazoline moiety, and the resulting **57** molecule exhibited acceptable BBB permeability, potent propidium iodide displacement from AChE’s CAS, and lack of neurotoxic effect on SH-SY5Y cells for the maximum concentration of 80 μM. In a dose-dependent manner, **57** inhibited self-induced (up to 56.11%) and AChE-induced (up to 62.88%) Aβ aggregation. Neuroprotective effects were also observed in Aβ_1–42_ treated cell lines. Confirmed tolerance for doses up to 500 mg/kg allowed the further evaluation of **57** in rats, revealing enhancement of cognitive function when administered to Aβ-induced animal models. Immunochemistry evaluation disclosed significant downregulation of BACE-1 and Aβ levels, while the hippocampal histopathology examination revealed increased unaltered neuronal cells. In the Drosophila model of AD, compound **57** successfully reversed the Aβ-induced eye phenotype. In silico experiments verified the previous findings, predicted good per os bioavailability, and no violation of Lipinski’s rule.

Singh et al. [70] reported another great series of oxadiazole-piperazine conjugates for potential inhibition of AChE, BACE-1, and Aβ aggregation. The most promising compound **58** is a selective AChE and BACE-1 inhibitor (AChE; IC_50_ = 0.103 μM, BACE-1; IC_50_ = 1.342 μM). Kinetic studies revealed that **58** could occupy both CAS and PAS of AChE. The PAMPA suggested acceptable BBB penetration. Additionally, **58** managed to inhibit self- and AChE-induced Aβ aggregation and demonstrated a strong percentage of radical scavenging (44.35%) in a dose-depended manner. Tolerance at doses up to 100 mg/kg was observed and, in a scopolamine-induced model the administration of **58** effectively enhanced spatial memory. The ex vivo testing of hippocampal tissue suggested a decrease in AChE and showcased antioxidant activity by examining the biomarkers of catalase and malonaldehyde. In silico evaluation estimated that **58** follows Lipinski’s rule of five and displays good oral absorption.

Banoo et al. [71] evaluated indole-piperidine amides against AChE, BuChE and BACE-1. The most advantageous compound **59** exhibited potent inhibition of AChE and BACE-1 (AChE; IC_50_ = 0.32 μM, BACE-1; IC_50_ = 0.39 μM), molecular docking studies supported the mixed-type activity findings. The PAMPA proposed significant BBB permeability, and the authors recommended that these findings provide a strong basis for further in vivo investigation.

Sharma et al. [72] explored novel indol-3-yl-phenyl allylidene hydrazine carboximidamide derivatives and the most potent compound **60** exhibited inhibitory activity against AChE and BACE-1 (AChE; IC_50_ = 60.93 μM, BACE-1; IC_50_ = 9.38 μM). Molecular modeling studies revealed the occupation of both CAS and PAS of AChE and in silico prediction favored drug-likeness and pharmacokinetic properties.

Khan et al. [73] employed in silico methods to screen compounds, carefully considering pharmacokinetics and BBB permeability. Molecule **61** was then thoroughly investigated through in vivo testing. The **61** consists of pyrrolopyridine and *N*-cyclohexyl moieties and interacts with BuChE, BACE-1, γ-secretase, MAO-A and MAO-B. The administration of **61** alone or in combination with conventional AD drugs like donepezil and memantine significantly improved locomotor activity, neuromuscular coordination issues, and memory deficits. **61** treatment not only enhanced cognitive and motor functions but also reduced neuroinflammation, preserved brain tissue morphology, and decreased lesion volumes.

**Table 7:** Potential multitargeted drugs for AD against secretases.

Molecule	Molecule Name	Chemical Class	Primary Targets
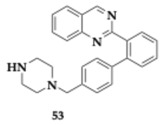	**AV-2** [69]	Quinazoline derivative	**AChE** (IC_50_ = 0.193 μM) **BuChE** (IC_50_ > 10 μM) **BACE-1** (IC_50_ = 0.254 μM) **Aβ self- induced aggregation inhibition** (29.18–48.72%) **Aβ AChE-incused aggregation inhibition** (60.38–69.00%)
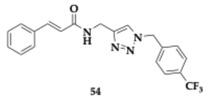	**4j** [66]	Cinnamamide/ester triazole hybrid	**Copper-induced Aβ toxicity inhibition** (EC_50_ = 1.39 μM) **AChE inhibition**
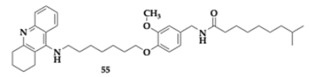	**5s** [67]	Capsaicin-tacrine hybrid	**AChE** (IC_50_ = 69.8 nM) **BuChE** (IC_50_ = 68.0 nM) **BACE-1** (IC_50_ = 3.6 μM)
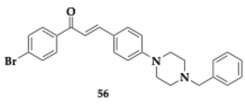	**41** [68]	*N*-aryl piperazine-chalcone hybrid	**AChE** (IC_50_ = 14.84 μM) **BuChE** (IC_50_ = 41.39 μM) **BACE-1 inhibition** (46.60%) **self- and AChE-induced Aβ_1–42_ aggregation inhibition**
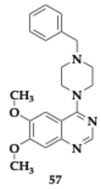	**AK-2** [69]	Piperazine-quinazoline hybrid	**AChE** (IC_50_ = 0.283 μM) **BACE-1** (IC_50_ = 0.231 μM) **self-induced Aβ aggregation inhibition** (56.11%) **AChE-induced Aβ aggregation inhibition** (62.88%)
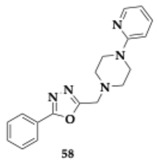	**5AD** [70]	Oxadiazole-piperazine conjugate	**AChE** (IC_50_ = 0.103 μM) **BACE-1** (IC_50_ = 1.342 μM) **Self- and AChE- induced Aβ aggregation inhibition** **Radical scavenging activity** (44.35%)
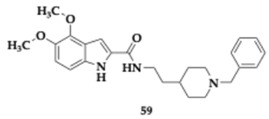	**23a** [71]	Indole-piperidine amide	**AChE** (IC_50_ = 0.32 μM) **BACE-1** (IC_50_ = 0.39 μM)
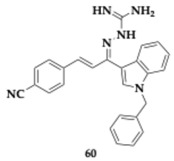	**1l** [72]	Indol-3-yl-phenyl allylidene hydrazine carboximidamide derivative	**AChE** (IC_50_ = 60.93 μM) **BACE-1** (IC_50_ = 9.38 μM)
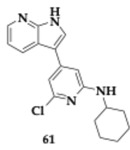	**SSZ** [73]	Pyrrolopyridine and *N*-cyclohexyl hybrid	**BuChE** **BACE-1** **γ-secretase** **MAO-A** **MAO-B**

### 2.8. Compounds Against MAO-B

Kumar et al. [74] evaluated the efficacy of chromone derivatives in the treatment of AD. In vitro testing revealed three possible candidates, and the most promising compound **62** (Table 8) exhibited balanced inhibitory activity against the targeted enzymes (AChE; IC_50_ = 140 nM, BuChE; IC_50_ = 11.6 μM, MAO-B; IC_50_ = 450 nM), managed to inhibit Aβ self-induced aggregation by 47.3%, reduced ROS, presented moderate antioxidant activity (68.44%), and cytocompatibility was evaluated for concentrations up to 25 μM at SH-SY5Y cells. Moreover, **62** was found to be a reversible inhibitor of AChE, and kinetic studies disclosed that all compounds were able to bind simultaneously to CAS and PAS. Further in vivo investigation conducted in a scopolamine-induced zebrafish model for **62** revealed similar effects compared to donepezil and improved cognitive function. The three leading compounds showed good oral bioavailability, BBB permeability, and no violation of Lipinski’s rule of five was observed.

Fan et al. [75] conducted a comprehensive evaluation of the safety of promising anti-AD MTDLs identified in their previous research. The authors’ primary argument is that most preclinical studies focus primarily on evaluating efficacy, often neglecting thorough safety assessments. The leading compound **63** demonstrated potent MAO-B inhibition, iron chelation, and provided neuroprotection against oxidative stress. Furthermore, it effectively ameliorated behavioral and cognitive function in scopolamine-induced mouse models of AD. This study demonstrated that **63** exhibits no acute oral toxicity at doses up to 2000 mg/kg and no genotoxic effects in various assays, including the Ames test, bone marrow micronucleus assay, and in vitro Chinese Hamster Lung Cell Assay (CHL assay). Additionally, no reproductive or embryotoxic effects were observed at doses up to 1000 mg/kg, and a 90-day oral toxicity study showed no adverse effects on physiological parameters at doses up to 500 mg/kg. No gender-based differences in toxicity were detected. These results indicate that **63** is safe for further investigation as a potent therapeutic agent against AD.

Al-Saad et al. [76] designed 6-hydroxybenzothiazol-2-carboxamide with potential activity against MAO-B, tau, and α-synuclein. The compound **64** includes a phenethyl side chain. It is capable of potent MAO-B inhibition (IC_50_ = 41 nM) and can effectively reduce tau and α-synuclein aggregation in vitro. Furthermore, **64** is considered safe at concentrations up to 100 μΜ, suggested by the cytotoxicity evaluation at SH-SY5Y cells, and exhibits significant neuroprotective effects comparable to estradiol. The PAMPA revealed good BBB penetration, and the authors suggested further optimization of **64,** considering the microsome stability.

### 2.9. Inhibition of Cholinesterases

Kilic et al. [77] designed eight new N′-(quinolin-4-ylmethylene)propanehydrazides as potent cholinesterase inhibitors, antioxidants, and metal chelators. The most balanced inhibitor was compound **65** (AChE; IC_50_ = 7.04 μM, BuChE; IC_50_ = 16.06 μM), although it was not the most potent (Table 9). The most active compound achieved an IC_50_ value of 0.69 μM regarding AChE activity, and the kinetic study revealed a mixed-type inhibition for both compounds. The antioxidant activity was found to be inversely related to the activity against cholinesterases, as the most active compounds presented decreased antioxidant ability. Metal binding studies assessed the ability of the compounds to chelate iron, copper, and zinc ions. In silico experimentation predicted acceptable BBB permeability and good drug-likeness.

Soliman et al. [78] designed and synthesized piperazine-2-carboxylic acid derivatives as MTDLs against AChE and BuChE. The most active compounds regarding each enzyme were compound **66** for AChE (Ki = 10.2 μM) and **67** for BuChE (Ki = 1.6 μM). The cytotoxicity evaluation for both compounds was conducted on SH-SY5Y cells, revealing that their toxic effects are close to those of donepezil and staurosporine. The SAR investigation showed the selectivity of the carboxylic acid series and piperazinyl-2-hydroxamic acid series towards AChE and BuChE, respectively. Carbohydrazide derivatives appeared to be less potent against both enzymes. Finally, a variety of possible substituents in the benzyl ring were examined. Nearly all tested compounds demonstrated zero violations of Lipinski’s rule and were expected to penetrate the brain effectively.

Tamaddon-Abibigloo et al. [79] introduced a series of fifteen isatin-triazine-aniline hybrids as potential anti-AD agents. The compound **68** exhibited strong inhibitory activity against key enzymes (AChE; IC_50_ = 0.2 nM, BuChE; IC_50_ = 0.03 μM), and from **69,** antioxidant properties were observed (EC_50_ = 64.4 μM). The most advantageous substitutions were the *n*-benzyl on isatin and the 2-OMe or 2-OH on aniline. Furthermore, **68** was able to chelate zinc, iron, and copper ions. Kinetic studies showed AChE competitive and BuChE non-competitive inhibition, while molecular docking studies revealed an interaction with both PAS and CAS of AChE.

Jevtić et al. [80] designed a series of tacrine derivatives as novel MTDLs by utilizing a five-methylene linker. The leading compound **70** has a 2-clorobenzoyl moiety and presents inhibitory activity against cholinesterases (AChE; IC_50_ = 156.0 nM, BuChE; IC_50_ = 82.5 nM). Molecular docking studies suggested interaction with both PAS and CAS of AChE. Molecule **70** did not exhibit cytotoxic effects against SH-SY5Y cell lines and demonstrated neuroprotective and antioxidant potential by H_2_O_2_-induced ROS. In silico studies resulted in a violation of Lipinski’s rule regarding molecular weight and predicted good BBB permeability.

In their study de Sousa et al. [81] investigated in silico multiple huperzine A hybrids. One of the leading compounds **71**, especially the S’R’ enantiomer, was able to inhibit AChE with significant free binding energy and managed to chelate metal ions such as zinc and especially iron, herein suggesting anti-ferroptosis effect in AD patients. Different pharmacological activities were observed among the enantiomeric conformations of the compounds. Moreover, good oral bioavailability and BBB penetration were predicted. Further investigations are needed for these anti-AD candidates.

Kumar et al. [82] designed novel MTDLs by exploring the piperic acid scaffold. The most promising molecule **72** managed to inhibit both cholinesterases (AChE; IC_50_ = 2.13 μM, BuChE; IC_50_ = 28.19 μM), could effectively displace propidium iodide from PAS and kinetic studies revealed mixed-type inhibition of AChE. Furthermore, antioxidant activity was observed, and no metal-chelating properties were displayed. The compound presented no violation of Lipinski’s rule, and PAMPA revealed sufficient BBB permeation. The cytocompatibility was monitored on SH-SY5Y cells, and **72** found to be well tolerated at doses up to 500 mg/kg; absence of histopathological findings in the liver and kidney. In vivo evaluation in scopolamine-induced models suggested advantageous activity of **72** regarding spatial memory and cognitive abilities.

Nagani et al. [83] explored piperazine-quinoline agents and the most promising hit, compound **73**, presented potent inhibition of AChE and BuChE (AChE; IC_50_ = 3.013 μM, BuChE; IC_50_ = 3.144 μM). Molecule **73** containing 4-methoxyphenyl and 4-chloroanilino moiety exhibited a high affinity against these enzymes, higher than donepezil. Molecular modeling studies revealed that **73** can interact with PAS and CAS of AChE. Antioxidant properties and metal chelating abilities were also observed. In silico evaluation suggested favorable pharmacokinetic properties and BBB permeability.

### 2.10. Antagonism of NMDA Receptors

Mezeiova et al. [84] attempted to create amiridine hybrids by linking various active scaffolds and therefore expanding the pharmacological effects of the novel compounds. Their main goal was to achieve NMDA antagonism (with memantine or adamantylamine moiety), antioxidant (with trolox moiety), and anti-amyloid activity (with substituted benzothiazole moiety), maintaining the dual AChE and BuChE inhibition. The most active inhibitors were **74** (IC_50_ = 0.6 μM) and **75** (IC_50_ = 0.1 μM) regarding BuChE (Table 10), the analogs containing memantine moiety exhibited more potent activity compared to adamantylamine counterparts and displayed equal potency with tacrine. Furthermore, more favorable inhibition of BuChE was observed when the amide part of the linker was placed closer to the aforementioned moieties. Compounds **74** and **75** demonstrated a low cytotoxic profile on SH-SY5Y cell lines, and the authors suggested further examination regarding the NMDA antagonism, improvement in solubility, and BBB permeability. Although molecules from the aziridine-benzothiazoles group displayed high BuChE inhibitory activity, toxicity, and no inhibition of Aβ self-induced aggregation were confirmed.

Misiachna et al. [85], in continuation of their prior work, explored thirty phenoxytacrine derivatives aiming to reduce the hepatotoxic effect. The leading agent **76** inhibited AChE (IC_50_ = 8.52 μM) and BuChE (IC_50_ = 5.48 μM) and managed to antagonize selectively the GluN1/GluN2B subtype of the NMDA receptor (IC_50_ = 2.4 μM), due to the interaction with the ifendprodil-binding site. The in vivo pharmacokinetic profile of **76** is sufficient. It presented favorable BBB penetration and was considered to be safe at doses up to 5 mg/kg. Additionally, **76** is predicted to have sufficient microsomal stability and no hepatotoxic biotransformation. Although the ability to inhibit in vivo AChE remains unclear, the administration of **76** in scopolamine-induced models enhanced cognitive function.

### 2.11. Inhibition of MAO and Carbonic Anhydrase

Giovannuzzi et al. [86] evaluated dual inhibitors of MAO-B and carbonic anhydrase (CA) by linking coumarin and chromone moieties with benzesulfonamide fragments. 15 human (h) carbonic anhydrase (CA) isoforms are located in regions of the brain involved in cognitive functions. These enzymes are essential for pH level regulation and ion transportation. Their inhibition is linked to a reduction in Aβ-induced production of ROS and mitochondrial dysfunction, and recent findings suggest the mitigating effects in neuronal apoptosis by the Food and Drug Administration (FDA)-approved CA inhibitors (acetazolamide and methazolamide). Herein, due to the synergistic dual inhibition, the hybrids were tested for their protective role in Aβ-induced neurotoxicity.

Specifically, the authors created four distinct categories by applying different substitutions and linkers on the pharmacophores and evaluated their ability to interact with the different CA and MAO isoforms. Each compound assessed exhibited selectivity for MAO-B (MAO-A; IC_50_ > 10 μM). Among the various subsets, 7-(4-sulfamoylbenzyl)oxycoumarin derivatives demonstrated strong inhibition regarding MAO-B and showed sufficient activity against diverse CA isoforms, including II, VII, and XII. Namely, the estimated IC_50_ value of compounds **77** and **78** regarding MAO-B is 9.1 and 6.7 nM, respectively (Table 11). Molecules **79** and **80** were identified as potent as compound **78**. From this article, valuable information can be obtained regarding various combinations of substituents and their corresponding activities. The neuroprotective properties of the compounds assessed on SH-SY5Y cell lines often exceed the reference drugs. Evaluation of the pharmacokinetic properties distinguished compound **78** as a possible candidate for in vivo studies and diminished **75** for poor membrane permeability [47].

### 2.12. Compounds Against Cholinesterases, Diabetes, Cancer, and Inflammation

Naglah et al. [87] searched for pyrazole-based Schiff bases for potential activity against AD, diabetes, inflammation, and carcinoma. Compound **81** (Table 12) presented excellent antioxidant, radical scavenging, and anti-inflammatory abilities. For the antioxidant and scavenging properties, the presence of substituents on the aromatic ring is essential. Additionally, due to the potent inhibition of α-amylase and α-glucosidase, **81** is considered a potential anti-diabetic candidate. Regarding anti-AD activity, **81** exhibited remarkable AChE inhibition (IC_50_ = 62.11 μM). In silico testing predicted sufficient BBB permeability and intestinal absorption. The cytotoxic evaluation revealed a high therapeutic index of **81** in the human colon carcinoma cell line (Caco-2 cells).

### 2.13. Compounds Against Ca^2+^ Channels, Nrf2 Pathway, Cathepsin S Enzyme, and Oxidation

Santos et al. [88] reported the first novel MTDLs against Ca^2+^ channels, Nuclear factor erythroid 2-related factor 2 (Nrf2) pathways, Cathepsin S (CatS) enzyme, and oxidation. In AD patients, increased calcium levels can interfere with mitochondrial function and cause cell death, Aβ, and tau proteins. In vitro experiments suggested the promotion of tau aggregation by the protease CatS, while overexpression of this enzyme is related to Aβ pathology. Finally, the activation of Kelch-like ECH-associated protein 1 (Keap1)-Nrf2- Antioxidant Response Element (ARE) signaling pathway is significant for antioxidant regulation. The leading compound **82** (Table 13) is able to block the calcium channel by 11% and presents strong antioxidant activity. No cytotoxic effects were observed at Antioxidant Response Element cell line 32 (AREc32 cells) for the maximum concentration (150 μM), and all tested compounds managed to induce the Nrf2 activation pathway (**82**; concentration (CD) = 96.0 μM). The results indicate important SAR information; substitution on the aromatic ring decreases antioxidant properties, and the ortho-chloro substituent of **82** is the most favorable regarding the Nrf2 activation. Additionally, compound **82** is able to interact with CatS with an estimated K_i_ value of 69.3 μΜ [88].

### 2.14. Inhibition of MAO and Xanthine Oxidase

D’Errico et al. [89] evaluated a series of hydroxytyrosol derivatives of donepezil as potential agents against neurodegenerative diseases, including AD and PD. Authors identified **83** (Table 14) as potent MAO-A and MAO-B inhibitors with an IC_50_ value of 23.4 μM and 171.0 μM, respectively. Compound **83** inhibits xanthine oxidase significantly, which regulates multiple redox species, and its stimulation has been associated with neurodegeneration. Regarding the SAR, the presence of a nitro group on **83** and **84** is associated with a lack of MAO selectivity. Additionally, acetylation of the tested compounds does not improve their activity; instead, it primarily serves to enhance membrane permeability.

## 3. Multitarget Compounds Against PD

### 3.1. Compounds with Neuroprotective Potential

Di Maio et al. [90] explored the neuroprotective activity of 10-nitro-oleic acid, compound **85** (Table 15), as an anti-PD agent. In dopaminergic neurons, hyperactivation of Nicotinamide Adenine Dinucleotide Phosphate (NADPH) oxidase isoform 2 (NOX2) and leucine-rich repeat kinase 2 (LRRK2) has been related to the pathogenesis of PD, while neuroprotection is observed upon Nrf2 activation. In rotenone-induced mouse midbrain dopaminergic neuronal cell lines (N27-A), compound **85** could avert oxidative stress and LRRK2 activity. In summary, the advantageous activity of **85** can be attributed to a reduction in oxidative stress, α-syn buildup, excessive NOX2 and LRRK2 activity, microglial response, and disruption of mitochondrial protein import.

Pan et al. [91] thoroughly investigated in vitro and in vivo compound **86** as a potential anti-PD agent. The authors predicted good brain permeability, low toxicity, and metabolism by Cytochrome P450 3A4 (CYP3A4). Neuroprotective effects of **86** were observed as mitochondrial membrane potential (Δψm) diminished in PC12 cells treated with a 6-hydroxydopamine (6-OHDA) neurotoxic agent. Moreover, **86** improved glycolytic activity, mitochondrial respiration, and biosynthesis. In vivo testing displayed its neuroprotective role, as it enhanced motor activity and successfully minimized the death of dopaminergic neurons in mice when administered at 3 mg/kg/day. Compound **86** interacts with two major pathways: cAMP Response Element-Binding Protein/Peroxisome Proliferator-Activated Receptor Gamma Coactivator 1-alpha/Nuclear Respiratory Factor 1/Transcription Factor A Mitochondria (CREB/PGC-1α/NRF-1/TFAM) and Protein Kinase A/Protein Kinase B/GSK-3β (PKA/Akt/GSK-3β).

Anastassova et al. [92] studied novel benzimidazole arylhydrazones and the highly promising compound **87** presented cytocompatibility in vitro and neuroprotection against H_2_O_2_-induced oxidative stress, superior to that of melatonin and rasagiline. In rat brain synaptosomes upon 6-OHDA-induced toxicity, treatment with **87** verified the neuroprotective potential of the compound. Further molecular docking investigation suggested significant inhibition of MAO-B. The compound **87**, characterized by a 2,3-dihydroxy substitution, demonstrated a significant protective effect against ferrous iron-induced oxidative damage in lecithin. Additionally, **87** exhibited strong antioxidant properties, including the ability to scavenge superoxide radicals and reduce deoxyribose oxidation.

**Table 15:** Potential multitargeted drugs for PD with neuroprotective activity.

Molecule	Molecule Name	Chemical Class	Primary Targets
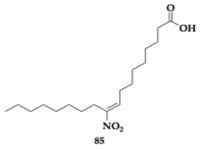	**NOA** [90]	10-nitro-oleic acid	**Oxidative stress** **α-syn** **NOX2 inhibition** **LRRK2 inhibition**
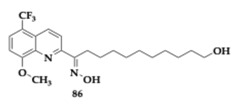	**J24335** [93]	Quinoline derivative	**CREB/PGC-1α/NRF-1/TFAM** **PKA/Akt/GSK-3β**
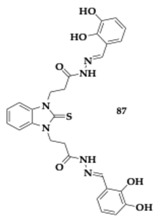	**3h** [94]	Benzimidazole arylhydrazone derivative	**Oxidative stress** **MAO-B inhibition**

### 3.2. Compounds Against Neuroinflammation

Albertini et al. [95] evaluated neflamapimod-rasagiline hybrids against Lewy body dementia, but due to the overlapping pathological mechanisms, their findings might be valuable regarding PD and AD treatment. Compound **88** (Table 16), a *N*-methyl-*N*-propargyl derivative, managed to inhibit potently p38 mitogen-activated protein kinase (p38α-MAPK) (IC_50_ = 98.7 nM), which is a kinase associated with neuroinflammation. Neuroprotective effects against 6-OHDA were not displayed, and no interaction with MAOs was found. Moreover, ROS production diminished in dexamethasone-induced SH-SY5Y cells, while the regulation of pro-inflammatory markers in microglia was exhibited. The authors suggested further optimization regarding the unfavorable toxicity and BBB penetration.

Wu et al. [96] designed and explored carbamylated tryptamine hybrids as anti-PD and anti-AD agents. Most of the evaluated compounds exhibited potent inhibition against BuChE. Molecule **89** significantly interacts with BuChE with an estimated IC_50_ of 6.77 nM. The **89** contains a dibenzyl-substituent and shows cytocompatibility in vitro, while displaying neuroprotective effects at H_2_O_2_ mouse hippocampal cell lines (HT-22 cell lines), at 5 μM concentration. In addition, authors reported inhibition of COX-2, and in silico evaluation revealed favorable BBB permeability.

Duarte et al. [97] evaluated an innovative series against PD by combining selective MAO-B inhibition and NRF2 induction. NRF2 regulates multiple cellular functions, and its downregulation is a significant risk factor for the development of PD. One of the most advantageous hits, compound **90**, induces NRF2 with an estimated CD value of 5.07 μM and inhibits MAO-B (IC_50_ = 17.0 μM). In addition, favorable BBB permeability was predicted by PAMPA, followed by an IL-1β reduction up to 41.1% (anti-inflammatory activity) and presented antioxidant potential, which was verified in SH-SY5Y cells. Molecule **90** upregulates NRF2-dependent proteins significant decrease in ROS production and reduction in cell death.

Guo et al. [98], based on their previous research on styryl sulfone MTDLs, explored further their potential. A promising candidate, compound **91**, exhibited inhibition of NO up to 94.0% in a dose-dependent manner and possessed antioxidative and anti-neuroinflammatory properties in PD models. Specifically, molecule **91,** which features a chloro-substitution, exhibited potent neuroprotective effects by inhibiting the p38 MAPK and NF-κB pathways, reducing neuroinflammation, and activating the Nrf2 pathway to combat oxidative stress.

### 3.3. Compounds Against MAO-B and Other Pathological Targets

Al-Saad et al. [76] tested novel 6-hydroxybenzothiazol-2-carboxamides against the main pathologic targets of PD and AD. The most promising hit, compound **92** (Table 17), inhibits selectively MAO-B (IC_50_ = 0.041 μM). Regarding the exploration of SAR, the increased linker length and the phenethyl side chain enhanced the activity. Furthermore, **92** inhibited tau and α-syn aggregation (tau; EC_50_ = 10.5 μM, α-syn; IC_50_ = 9.09 μM). Compound **92** demonstrated multipotent activity in SH-SY5Y cells, showing cytocompatibility at concentrations up to 100 μM and providing neuroprotection against toxicity induced by both tau and α-synuclein. The PAMPA revealed high BBB penetration, while good pharmacokinetic properties were predicted. The authors suggest further optimization regarding metabolic stability.

Chavarria et al. [99] designed potential anti-PD agents by exploring caffeic acid derivatives. One of the most promising derivatives is compound **93**, able to inhibit membrane-bound COMT -the main isoform located in the brain- and MAO-B (COMT; IC_50_ = 1.33 μM, MAO-B; IC_50_ = 4.27 μM). Additionally, **93** exhibited significant radical scavenging ability. Cytotoxic evaluation conducted on SH-SY5Y cells suggested safety for doses up to 50 μM, but at 100 μM, neurotoxic effects appeared due to the formation of ROS. The PAMPA demonstrated favorable brain penetration for compound **93**.

Anastassova et al. [94] synthesized and tested benzimidazole hybrids as potential multitarget PD’s therapeutics. The leading hit **94**, a substituted arylhydrazone, presented negligible cytotoxicity in SH-SY5Y cells (IC_50_ > 250 μM) and rat brain synaptosomes. In vitro evaluation suggested neuroprotective potential against H_2_O_2_-induced oxidative damage. Potent inhibitory activity of **94** against MAO-B was also observed. In a lecithin-induced lipid peroxidation model, compound **94** effectively mitigated ferrous iron-induced oxidative damage.

Elsherbeny et al. [100] designed MTDLs against PD by utilizing an indole scaffold. Molecule **95** inhibits selectively MAO-B (IC_50_ = 0.02 μM), and its kinetic studies suggested a competitive activity mode. An essential point related to MAO-B selectivity is the free-NH group and the substitution of indole, driving a crucial hydrogen bond with the enzyme. Cytotoxic evaluation conducted on PC12 cells and revealed safety of **95** up to 30 μM concentration. Additionally, compound **95** exhibited substantial neuroprotective effects in cell lines exposed to 6-OHDA and rotenone, while also significantly reducing ROS production.

Elkamhawy et al. [101] synthesized and evaluated twenty-four melatonin hybrids against PD. Among the tested compounds, **96** is a promising hit able to selectively inhibit MAO-B (IC_50_ = 1.41 μM), displayed in silico favorable BBB permeability, and compatibility with Lipinski’s rule. Regarding SAR, it was mainly observed that substituting the benzamide ring at the meta and para positions resulted in a significant enhancement of inhibitory activity against MAO-B. Cytocompatibility up to 30 μΜ concentration of **96** was found in PC12 cells, while the neuroprotective potential was observed on 6-OHDA- and rotenone-induced toxicity; **96** increased cell viability up to 66.4% and 82.7%, respectively.

### 3.4. Compounds Against Cholinesterases, MAOs, and Other Pathological Targets

Berrino et al. [102] reported another series of potential CA, MAO, and cholinesterase inhibitors by designing alkyl-substituted coumarins. Among the most promising hits, compound **97** (Table 18) demonstrated potent inhibitory activity against CA isoforms VII, IX, and XII, in the nanomolar range. Furthermore, **97** exhibited significant inhibition of MAO-B (IC_50_ = 0.007 μM), and kinetic studies also revealed interaction with MAO-A. Additionally, favorable inhibition of AchE and BuChE was displayed, and in silico evaluation suggested good BBB permeation. Reduction in pro-inflammatory cytokines observed during cellular assays, as well as decreased levels of lipopolysaccharide (LPS)-induced generation of H_2_O_2_.

Vicente-Zurdo et al. [103] evaluated novel rivastigmine-benzimidazole MTDLs against PD and AD. These hybrids were able to inhibit cholinesterases, Aβ aggregation, and MAOs, antioxidant and metal chelating capacity. One of the most significant compounds, **98**, was able to chelate copper ions and inhibited both self- and Cu-induced Aβ_42_ aggregation by approximately 45%. Interaction with MAO-A and MAO-B was also observed. In vivo evaluation shows the neuroprotective abilities of **98** in the PD cell model. The SAR revealed that the cholinesterase inhibitory effect is mainly related to the rivastigmine moiety, whereas the benzimidazole moiety and its inherent substituents, particularly those with metal-chelating properties, are chiefly responsible for the anti-Aβ aggregation and antioxidant activities.

Naseem et al. [104] synthesized 1,3,4-oxadiazoles as anti-PD and anti-AD agents. Among the tested compounds, promising inhibitory activity against cholinesterases and MAOs observed. For example, **99** is a potent multitarget inhibitor (AChE; IC_50_ = 0.83 μM, MAO-A; IC_50_ = 1.44 μM, MAO-B; IC_50_ = 1.04 μM), and is computationally predicted to effectively penetrate BBB.

Kulikova et al. [105] utilized a 1H-chromeno[3,2-c]pyridine scaffold with activity against PD, AD, and potential anticancer ability. Recent findings suggest that the inhibition of MAOs may be advantageous regarding the downregulation of tumor development and progression. Among the twenty synthesized compounds, **100** exhibited multipotent inhibitory effects (AChE; IC_50_ = 6.79 μM, MAO-A; IC_50_ = 8.42 μM, MAO-B; IC_50_ = 0.510 μM). The antiproliferative activity of **101** was verified in the Michigan Cancer Foundation breast cancer cell line (MCF-7), human colon cancer cell line 116 (HCT116), and Sankyo Ovarian 3 human ovarian adenocarcinoma (SK-OV-3) cell lines, highlighting a potential new candidate for cancer therapy.

Kamecki et al. [106] synthesized twenty-five novel 2′-hydroxychalcones as potential multipotent therapeutic agents against PD and AD. One of the most promising derivatives **102**, exhibited selective and reversible MAO-B inhibition with an IC_50_ value of 0.111 μM, while PAMPA suggested sufficient BBB penetration. In vitro evaluation demonstrated inhibitory activity against Aβ_1–42_ aggregation up to 75.7% at 10 μM concentration. The inhibition of AChE at the micromolar range and the in vivo experiments showcased high affinity against the benzodiazepine binding site of the γ-aminobutyric acid A (GABA_A_) receptors (Ki = 5.0 μM); herein, **102** displayed sedative and/or anxiety-reducing effects in mice.

Rodríguez-Enríquez et al. [107] utilized a 7-amidocoumarin scaffold and created hybrids against PD and AD. The authors synthesized sixteen molecules that inhibit MAO-A, MAO-B, AChE, BuChE, and BACE-1. The findings indicate that altering the substitutions at position 7 of the scaffold can lead to the development of different selectivity and multitarget profiles; for instance, compound **103** is a selective reversible MAO-B inhibitor (IC_50_ = 0.31 μM) with favorable pharmacokinetic properties. The cytocompatibility was exhibited among the synthesized compounds, but a lack of neuroprotective role was observed. A possible reason was suggested as the absence of hydroxyl groups.

### 3.5. Compounds Against Alpha-Synuclein (α-Syn) and Other Pathological Targets

Ramirez et al. [108] designed and tested eleven *N*- and *O*-linked indole triazines against major pathologic elements of PD and AD. The authors reported compound **104** as a promising scaffold that is worth future optimization (Table 19). The molecule **104** is an *N*-substituted triazine derivative, able to potently inhibit α-syn aggregation, with an estimated Fluorescence Intensity (FI) value of 13.5%. Furthermore, at the micromolar scale, **104** inhibits tau and hyperphosphorylated tau oligomerization, while ex vivo evaluation revealed the Aβ disaggregation activity.

Li et al. [109] investigated the role of pyrroloquinoline quinone, compound **105**, as a potential anti-PD MTDL. Thorough experimentation suggested antioxidant properties of **105** against the α-syn-119-induced generation of ROS in the presence of copper (II) ions, even in the molecular ratio of 1:4 α-syn to **105**. Furthermore, changes were observed to the secondary structure of α-syn while interacting with **105**, leading to inhibition of Cu-induced aggregation of α-syn.

Aboushady et al. [110] reported that compound **106**, derived from the *N*-phenethylbenzamide modification of the hydroxybenzothiazole urea scaffold, demonstrated significant multitarget activity against PD and AD. The study found that **106** not only selectively inhibited Dyrk1A, a key kinase associated with neurodegeneration, but also effectively blocked the aggregation of α-syn and tau proteins. Notably, **106** provided protection against neurotoxicity in SH-SY5Y cells induced by α-syn, tau aggregation, and 6-OHDA. The findings suggest that the simultaneous targeting of these multiple pathways by **106** results in enhanced neuroprotective effects compared to single-target agents. The authors concluded that further optimization is needed. However, the multitarget profile of **106** represents a promising candidate for developing more effective treatments.

### 3.6. Agonism of Dopamine Receptor and Antagonism of Adenosine Receptor

Kampen et al. [111] explored the sites of adenosine A2A receptor (A_2A_R) and dopamine receptor (D_2_R) to identify potential targets for future MTDLs. Authors utilized structure-guided design and reported compounds with affinity for these targets, able to act as D_2_R agonists. For instance, compound **107** presented K_i_ values of 0.16 and 0.37 μΜ against A_2A_AR and D_2_R respectively (Table 20). In vitro evaluation suggested good brain penetration.

### 3.7. Agonism of Histamine Receptor and Antagonism of Adenosine Receptor

Hagenow et al. [112] combined adenosine receptor antagonism with histamine receptor agonism and developed MTDLs as anti-PD agents. Specifically, the developed molecules targeted A_1_R, A_2A_R, H_3_R, and one of the most promising candidates, compound **108** exhibited high affinity with Ki values in the nanomolar range, especially against A_1_R/A_2A_R (Table 21). When molecule **108** was administered orally at 2 mg/kg, the wakefulness in mice was enhanced.

## 4. Multitarget Compounds Against HD

Although HD is a well-known neurodegenerative disorder, it has not yet garnered the same level of specific research attention as other conditions, such as AD and PD, in the context of multi-target compounds. Although recent studies have significantly advanced our understanding of MTDLs for neurodegenerative diseases in general, there remains a relative scarcity of focused research on HD-specific therapeutic strategies. Given the shared pathological features across these diseases, including protein misfolding, oxidative stress, and neuroinflammation, we can leverage insights from broader neurodegenerative studies. By identifying common molecular targets, it is possible to design MTDLs that could be applicable to HD, address multiple disease mechanisms, and advance therapeutic development for this underexplored condition.

In this context, Mousavi et al. [113] synthesized novel 3-aryl-5,6-dihydrobenzo[h]cinnolines as MTDLs to combat neurodegenerative diseases, including HD. Authors evaluated in silico the inhibitory activity of the characterized compounds against multiple pathological targets, such as AChE, MAO-A, BACE-1, and NMDA receptors. Among the leading molecules, **109** and **110** interacted with LIM-domain-containing protein kinase 2 (LIMK-2) and dihydroorotate dehydrogenase (DHODH) enzymes, respectively (Table 22). Specifically, molecular docking revealed a binding energy of -10.9 kcal/mol for **109** against LIMK-2 and -11 kcal/mol for **110** against DHODH. LIMK-2 regulates the actin cytoskeleton and multiple cell functions. DHODH is a mitochondrial enzyme that catalyzes a key step in the de novo biosynthesis of pyrimidine. Recent findings suggest the involvement of both enzymes in neurodegeneration and cancer. Additionally, authors reported that as anti-HD potential targets, c-Jun *N*-terminal kinases (JNKs) and *N*-Methyl-D-Aspartate Receptor (NMDAR), compound **110** exhibited favorable binding energies with both.

Jena et al. [114] evaluated quinoline hybrids containing a polyheterocycle scaffold as potent multi-target agents against neurodegeneration in HD and PD. The tested molecules inhibited cholinesterases and acted as cannabinoid receptor CB2 agonists. The molecules provide neuroprotective benefits through anti-inflammatory mechanisms without inducing psychoactive effects. For the synthesized compounds assessed in HD and PD models in vitro, molecule **111** stands out as one of the most promising quinoline derivatives reported in this article. It exhibits potent inhibition of BuChE, notable anti-apoptotic properties, effective reduction in mitochondrial superoxide radicals, and a restoration of mitochondrial membrane potential, making it an excellent candidate for further development.

Simmons et al. [115] assessed in vivo the therapeutic potential of compound **112** against HD, able to activate tropomyosin receptor-kinases (TrkB and TrkC). Based on previous research, the authors evaluated whether the co-activation of these two enzymes can result in synergistic activity and prevent neurodegeneration. The findings of the study indicate that **112** effectively activated TrkB and TrkC receptors in the striatum of R6/2 mice and normalized their signaling pathways. The compound significantly reduced intranuclear huntingtin aggregates and mitigated the degeneration of parvalbuminergic interneurons. It also decreased striatal inflammation, prevented the loss of dendritic spines and DARPP-32 in medium spiny neurons, and prevented motor deficits in HD mouse models. Additionally, microglial cell line LM22B-10 enhanced the phosphorylation of TrkB and TrkC at specific sites and activated AKT signaling, which resulted in increased phosphorylation of S6 at particular sites.

Załuski et al. [116] synthesized and evaluated nineteen xanthine-dopamine derivatives against neurodegenerative diseases, including HD, AD, and PD. The main targets of the tested compounds were the inhibition of MAO-B, antagonism of A_2A_ adenosine receptor (A_2A_AR), inhibition of phosphodiesterases -4 and -10 (PDE4, PDE10), and agonism of dopamine D_2_ receptor (D_2_R). Regarding AD and PD, the antagonistic activity against A_2A_AR induces D_2_R signaling and presents neuroprotection by decreasing oxidative damage, glutamate-induced neurotoxicity, and microglial response; in vivo experiments confirmed the favorable properties. Additionally, PDE4 is related to memory and cognitive function, and PDE10 has already been explored as an anti-HD candidate. Finally, the agonism of D_2_R palliates motor impairment and regulates neuroinflammation.

The authors reported a series of potent MAO-B inhibitors; among these compounds, **113** was able to interact with both MAO-B and A_2A_AR (MAO-B; IC_50_ = 47.9 nM, A_2A_AR; Ki = 0.672 μM). A promising compound regarding dual PDE inhibition is **114** (PDE4B1; IC_50_ = 2.44 μM, PDE10A; IC_50_ = 2.30 μM); short carbon chain and N7-benzyl or N7-phenylether substituents are essential for PDE activity. Some compounds even exhibited neuroprotective and antioxidant activity. Overall, Załuski et al. presented a promising starting point for targeting MAO-B, PDE, and adenosine receptors, which justifies further exploration [116].

## 5. Conclusions

Recently, medicinal chemists have hardly worked in the field of design and synthesis of multitarget agents to address neurodegenerative diseases such as AD, PD, and HD. Their research is trying to target the multifactorial pathogenesis of these diseases. Their derivatives are primarily intended to interrupt the sickness development and severity, imparting a higher quality of life. AD has been centralized in the research for the discovery of drugs to face neurodegeneration, since the number of AD patients is significantly higher compared to the PD and HD patients. As we have found reviewing the literature, HD especially has lower interest from medicinal chemists. Regarding the complexity of neurodegeneration, the multifunctional approach offers an alternative and potentially inventive manner of novel bioactive chemical entities.

Following this theory, a single molecule can be successful in behaving in a simultaneous modulation of various biological targets, with better ADMET properties and efficient cure. The molecular hybridization approach leads to hybrids with significant drug- ability and therapeutic results.

A perusal of the review titles and contents points to the fact that inhibition of cholinesterase combined with Aβ aggregation or histamine antagonism, or MAO inhibition led to potent AD’s and PD treatment. Secretases inhibition as well as inhibition of MAO and xanthine oxidase, agonism of dopamine receptor, and antagonism of adenosine receptor, agonism of histamine receptor, and antagonism of adenosine receptor target different biochemical pathways in AD, PD, and HD. Inflammation-related enzymes are consistently present in certain regions of the brain; in AD patients, elevated translation of LOX and COX2 is observed, leading to Aβ production. Thus, anti-inflammatory hybrids in combination with cholinesterase inhibition could offer treatment for AD, PD, and HD. Furthermore, antagonism of NMDA receptors and combined inhibition of MAO and carbonic anhydrase could cause complex health situations like diabetes, cancer, and inflammation. Multitarget compounds against Ca^2+^ channels, Nrf2 pathway, Cathepsin S enzyme, and oxidation offer the AD΄s treatment.

However, identifying novel agents capable of effectively modulating the progression of these devastating pathologies remains a significant challenge.

## Figures and Tables

**Table 1:** Potential multitargeted drugs for AD, inhibiting cholinesterases, and Aβ aggregation.

Molecule	Molecule Name	Chemical Class	Primary Targets
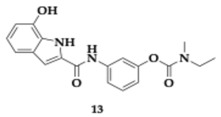	**5a3** [36]	Rivastigmine-indole hybrid	**AChE** (IC_50_ = 10.9 μM) **BuChE** (IC_50_ = 10.4 μM) **Aβ_42_ self-aggregation inhibition** (50.3%) **Antioxidant activity** (EC_50_ = 14.5 μM)
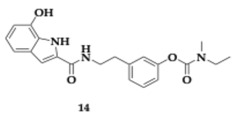	**5c3** [36]	Rivastigmine-indole hybrid	**AChE** (IC_50_ = 26.8 μM) **BuChE** (IC_50_ = 14.9 μM) **Aβ_42_ self-aggregation inhibition** (55.5%) **Antioxidant activity** (EC_50_ = 20.7 μM)
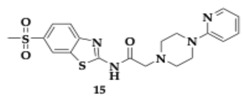	**1** [37]	Benzothiazole-piperazine hybrid	**AChE** (IC_50_ = 0.42 μM) **Aβ_1–42_ self-aggregation inhibition** (80.7%)
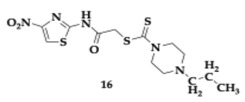	**10** [38]	Thiazole-clubbed piperazine derivative	**AChE** (IC_50_ = 0.151 μM) **BuChE** (IC_50_ = 0.135 μM) **Aβ_1–42_ self-aggregation inhibition** (73.53%)
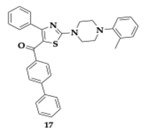	**20** [38]	Thiazole-clubbed piperazine derivative	**AChE** (IC_50_ = 0.499 μM) **BuChE** (IC_50_ = 0.103 μM) **Aβ_1–42_ self-aggregation inhibition** (79.42%)
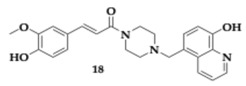	**13α** [39]	Ferulic acid-piperazine derivative	**AChE** (IC_50_ = 0.59 μM) **BuChE** (IC_50_ = 5.02 μM) **Antioxidant activity** (IC_50_ = 5.88 μM) **Aβ metal-induced and self-aggregation inhibition**
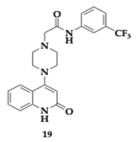	**AM5** [40]	Quinolinone hybrid	**AChE** (IC_50_ = 1.29 μM) **Aβ_42_ aggregation inhibition** (IC_50_ = 4.93 μM) **Antioxidant activity**
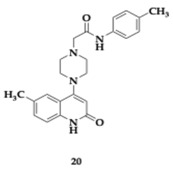	**AM10** [40]	Quinolinone hybrid	**AChE** (IC_50_ = 1.72 μM) **Aβ_42_ aggregation inhibition** (IC_50_ = 1.42 μM) **Antioxidant activity**
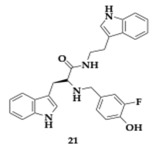	**3b-1** [41]	L-tryptophan derivative	**BuChE** (IC_50_ = 0.44 μM) **Aβ_1–42_ self-aggregation inhibition** (52.50%) **Radical scavenging** (IC_50_ = 0.72 μM)
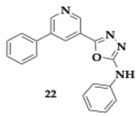	**5a** [42]	5-substituted-2-anilino-1,3,4-oxadiazole derivative	**AChE** (IC_50_ = 46.9 nM) **BuChE** (IC_50_ = 3.5 nM) **Aβ self-aggregation inhibition** **Antioxidant activity**
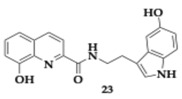	**6b** [43]	Melatonin–mydroxyquinoline hybrid	**Aβ Cu-induced and self-aggregation inhibition** (63.24%) **Radical scavenging**
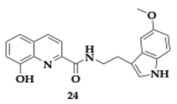	**6c** [43]	Melatonin–mydroxyquinoline hybrid	**Aβ Cu-induced and self-aggregation inhibition** (40.33%) **Radical scavenging**

**Table 2:** Potential multitargeted drugs for AD inhibiting GSK-3β.

Molecule	Molecule Name	Chemical Class	Primary Targets
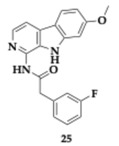	**ZLQH-5** [44]	Harmine derivative	**GSK-3β** (IC_50_ = 66 nM) **DYRK1A** (IC_50_ = 111 nM) **tau hyperphosphorylation inhibition**
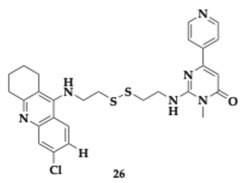	**18a** [45]	Tetrahydroacridin hybrid with sulfur-inserted linker	**GSK-3β** (IC_50_ = 0.930 μM) **AChE** (IC_50_ = 0.047 μM)
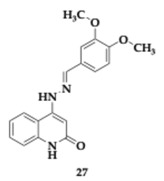	**7c** [46]	Quianolin-2-one derivative	**GSK-3β** (IC_50_ = 6.68 nM) **tau aggregation inhibition**

**Table 3:** Potential multitargeted drugs for AD inhibiting HDAC.

Molecule	Molecule Name	Chemical Class	Primary Targets
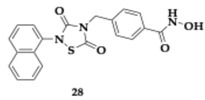	**4** [47]	Benzothiazepine derivative	**GSK-3β** (IC_50_ = 0.142 μM) **HDAC2** (IC_50_ = 0.030 μM) **HDAC6** (IC_50_ = 0.045 μM)
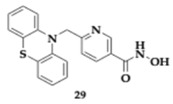	**W5** [48]	Carboxamidoxime derivative	**HDAC6 inhibition**
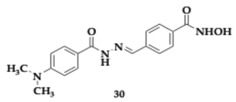	**LASSBio-1911** [49]	Benzohydrazide hybrid	**HDAC inhibition**

**Table 4:** Potential multitargeted drugs for AD inhibiting cholinesterases and presenting histamine antagonism.

Molecule	Molecule Name	Chemical Class	Primary Targets
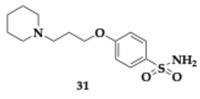	**2** [50]	Pitolisant-sulfonylureas derivative	**AChE** (IC_50_ = 7.65 μM) **H_3_R antagonism** (IC50 = 0.13 μM)
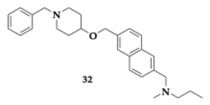	**ADS031** [51]	4-oxypiperidine ether derivatives	**H_3_R** (Ki = 12.5 nΜ) **AChE** (IC_50_ = 1.537 μM) **BuChE** (IC_50_ = 1.353 μM)
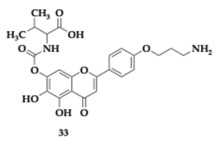	**11l** [52]	Scutellarein 7 L-amino acid carbamate-4′-cycloalkylamine propyl ether conjugate	**AChE** (IC_50_ = 9.73 μM) **H_3_R** (IC_50_ = 1.09 nM)

**Table 5:** Potential multitargeted drugs for AD against neuroinflammation.

Molecule	Molecule Name	Chemical Class	Primary Targets
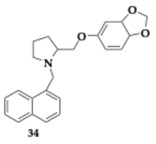	**7a** [53]	*N*-substituted pyrrolidine derivative	**AChE** (IC_50_ = 0.215 μM) **FAAH** (IC_50_ = 8.2 μM) **Antioxidant activity** (IC_50_ = 0.4 μM)
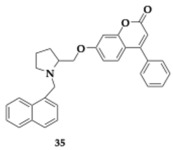	**14g** [53]	*N*-substituted pyrrolidine derivative	**BuChE** (IC_50_ = 0.155 μM) **FAAH** (IC_50_ = 0.94 μM) **Antioxidant activity** (IC_50_ = 42.7 μM)
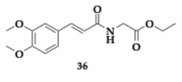	**18a** [54]	Cinnamic amide hybrid	**Lipid peroxidation inhibition** (99%)
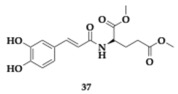	**28c** [54]	Cinnamic amide hybrid	**LOX-1** (IC_50_ = 8.5 μM)
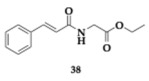	**22a** [54]	Cinnamic amide hybrid	**COX-2** (IC_50_ = 5 μM)
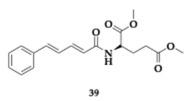	**23c** [54]	Cinnamic amide hybrid	**LOX-1** **COX-2**
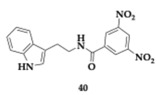	**SR42** [55]	Tryptamine derivative	**AChE** (IC_50_ = 0.70 μM) **MAO-B** (IC_50_ = 43.21 μM) **COX-2** (75.16%)
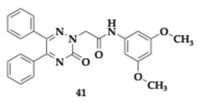	**S-12** [56]	Diaryl triazine hybrid	**AChE** (IC_50_ = 0.486 μM) **BACE-1** (IC_50_ = 0.542 μM) **Aβ_1–42_ aggregation inhibition** (81.1%)
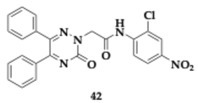	**S-02** [56]	Diaryl triazine hybrid	**DYRK1A** (IC_50_ = 2 μM)
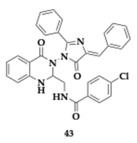	**7b** [57]	Quinazolinone-based hybrid	**AChE** inhibition (23.8%) **anti-inflammatory** **antioxidant activity**
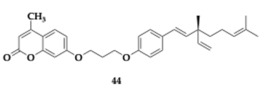	**3n** [58]	Bakuchiol–coumarin hybrid	**AChE** (IC_50_ = 34.78 μM) **TNF-α** (53.89%) **IL-6** (86.53%)
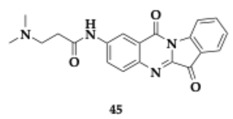	**n1** [59]	Tryptanthrin hybrid	**AChE** (IC_50_ = 12.17 nM) **Aβ_1–42_ aggregation inhibition** **NO** (IC_50_ = 2.13 μM) **IL-1β** (IC_50_ = 2.21 μM) **TNF-α** (IC_50_ = 2.47 μM)

**Table 6:** Potential multitargeted drugs for AD against cholinesterases and MAOs.

Molecule	Molecule Name	Chemical Class	Primary Targets
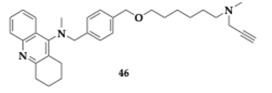	**7d** [60]	Tacrine-selegiline hybrid	**AChE** (IC_50_ = 1.57 μM) **BuChE** (IC_50_ = 0.43 μM) **MAO-A** (IC_50_ = 2.3 μM) **MAO-B** (IC_50_ = 4.75 μM)
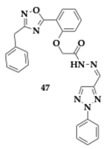	**3b** [61]	1,2,4-oxadiazole derivative	**AChE** (IC_50_ = 0.02330 μM) **MAO-B** (IC_50_ = 140.02 μM) **Antioxidant activity** (IC_50_ = 536.83 μM)
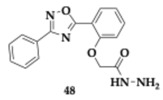	**1a** [61]	1,2,4-oxadiazole derivative	**MAO-A** (IC_50_ = 47.25 μM)
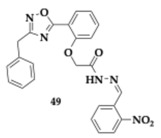	**2b** [61]	1,2,4-oxadiazole derivative	**AChE** (IC_50_ = 0.03393 μM)
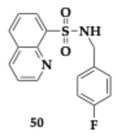	**a12** [62]	Quinoline-base sulfonamide hybrid	**AChE** (IC_50_ = 2.65 μM) **BuChE** (IC_50_ = 1.16 μM) **MAO-A** (IC_50_ = 1.34 μM) **MAO-B** (IC_50_ = 0.47 μM)
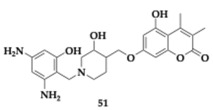	**4kk** [63]	Chromen-2-one based piperidine hybrid	**AChE** **BuChE** **MAO-A** **MAO-B**
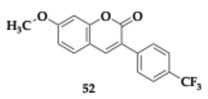	**5a** [64]	3-phenylcoumarin derivative	**AChE** **BuChE** **MAO-A** **MAO-B**

**Table 8:** Potential multitargeted drugs for AD against MAO-B.

Molecule	Molecule Name	Chemical Class	Primary Targets
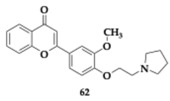	**VN-19** [74]	Chromone derivative	**AChE** (IC_50_ = 140 nM) **BuChE** (IC_50_ = 11.6 μM) **MAO-B** (IC_50_ = 450 nM) **Aβ self-induced aggregation** (47.3%) **Antioxidant activity** (68.44%)
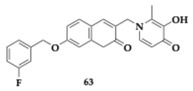	**XYY-CP1106** [75]	Hydroxypyridinone-coumarin hybrid	**Iron chelation** (pFe^3+^ = 18.04) **MAO-B** (IC_50_ = 14.7 nM)
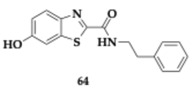	**30** [76]	6-hydroxybenzothiazol-2-carboxamid derivative	**MAO-B** (IC_50_ = 41 nM) **tau and α-synuclein aggregation reduction**

**Table 9:** Potential multitargeted drugs for AD inhibiting cholinesterases.

Molecule	Molecule Name	Chemical Class	Primary Targets
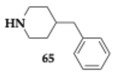	**4h** [77]	*N*′-(quinolin-4-ylmethylene)propanehydrazide hybrid	**AChE** (IC_50_ = 7.04 μM) **BuChE** (IC_50_ = 16.06 μM)
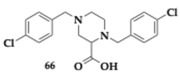	**4c** [78]	Piperazine-2-carboxylic acid derivative	**AChE** (Ki = 10.2 μM)
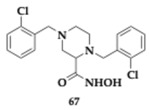	**7b** [78]	Piperazine-2-carboxylic acid derivative	**BuChE** (Ki = 1.6 μM)
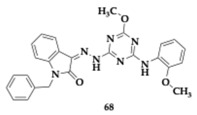	**8n** [79]	Isatin-triazine-aniline hybrid	**AChE** (IC_50_ = 0.2 nM) **BuChE** (IC_50_ = 0.03 μM) **Metal chelation**
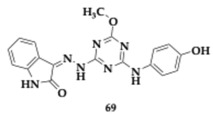	**8c** [79]	Isatin-triazine-aniline hybrid	**AChE** **BuChE** **Antioxidant activity** (EC_50_ = 64.4 μM)
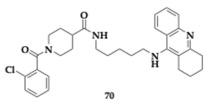	**16** [80]	Tacrine derivative	**AChE** (IC_50_ = 156.0 nM) **BuChE** (IC_50_ = 82.5 nM)
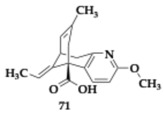	**HupA-A1** [81]	Huperzine A hybrid	**AChE inhibition** **Metal chelation**
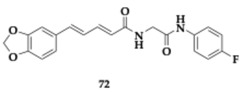	**6j** [82]	Piperic acid derivative	**AChE** (IC_50_ = 2.13 μM) **BuChE** (IC_50_ = 28.19 μM) **Antioxidant activity**
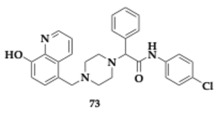	**95** [83]	Piperazine-quinoline hybrid	**AChE** (IC_50_ = 3.013 μM) **BuChE** (IC_50_ = 3.144 μM) **Antioxidant activity** **Metal chelation**

**Table 10:** Potential multitargeted drugs for AD presenting antagonism of NMDA receptors.

Molecule	Molecule Name	Chemical Class	Primary Targets
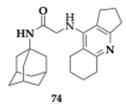	**5c** [84]	Amiridine hybrid	**BuChE** (IC_50_ = 0.6 μM) **NMDA antagonism**
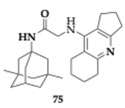	**5d** [84]	Amiridine hybrid	**BuChE** (IC_50_ = 0.1 μM) **NMDA antagonism**
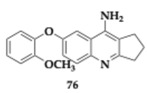	**I-52** [85]	Phenoxytacrine derivative	**AChE** (IC_50_ = 8.52 μM) **BuChE** (IC_50_ = 5.48 μM) **NMDA receptor antagonism** (IC_50_ = 2.4 μM)

**Table 11:** Potential multitargeted drugs for AD inhibiting MAO and carbonic anhydrase.

Molecule	Molecule Name	Chemical Class	Primary Targets
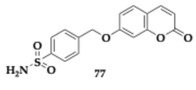	**45** [86]	Coumarin-benzesulfonamide hybrid	**MAO-B** (IC_50_ = 9.1 nM) **CA** (Ki = 0.1–90.0 nM)
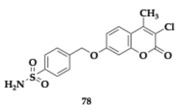	**47** [86]	Coumarin-benzesulfonamide hybrid	**MAO-B** (IC_50_ = 6.7 nM) **CA** (Ki = 0.1–90.0 nM)
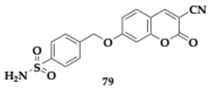	**75** [86]	Coumarin-benzesulfonamide hybrid	**MAO-B** (IC_50_ = 6.7–32.6 nM) **CA** (Ki = 0.1–90.0 nM)
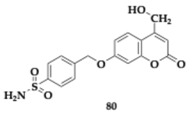	**84** [86]	Coumarin-benzesulfonamide hybrid	**MAO-B** (IC_50_ = 6.7–32.6 nM) **CA** (Ki = 0.1–90.0 nM)

**Table 12:** Potential multitargeted drugs for AD against cholinesterases, diabetes, cancer, and inflammation.

Molecule	Molecule Name	Chemical Class	Primary Targets
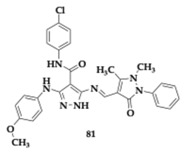	**7f** [87]	Pyrazole-based Schiff bases hybrid	**AChE** (IC_50_ = 62.11 μM) **α-amylase inhibition** **α-glucosidase inhibition**

**Table 13:** Potential multitargeted drugs for AD against Ca^2+^ channels, Nrf2 pathway, Cathepsin S enzyme, and oxidation.

Molecule	Molecule Name	Chemical Class	Primary Targets
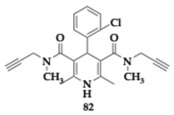	**4i** [88]	Dihydropyridine derivative	**Ca-channel blockage** (11%) **Induce Nrf2 activation** **Antioxidant activity** **CatS** (Ki = 69.3 μΜ)

**Table 14:** Potential multitargeted drugs for AD inhibiting MAO and xanthine oxidase.

Molecule	Molecule Name	Chemical Class	Primary Targets
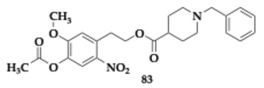	**HT3** [89]	Hydroxytyrosol derivatives of donepezil	**MAO-A** (IC_50_ = 23.4 μM) **MAO-B** (IC_50_ = 171.0 μM) **Xanthine oxidase inhibition**
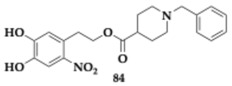	**HT2** [89]	Hydroxytyrosol derivatives of donepezil	**MAO-A inhibition** **MAO-B inhibition** **Xanthine oxidase inhibition**

**Table 16:** Potential multitargeted drugs for PD against neuroinflammation.

Molecule	Molecule Name	Chemical Class	Primary Targets
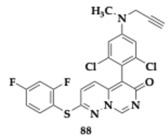	**4** [5]	*N*-methyl-*N*-propargyl derivative	**p38α-MAPK** (IC_50_ = 98.7 nM)
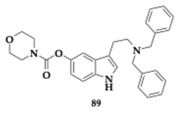	**2j** [96]	Carbamylated tryptamine hybrid	**BuChE** (IC_50_ = 6.77 nM) **COX-2 inhibition**
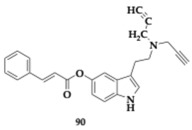	**14** [97]	Indole derivative	**NRF2** (CD = 5.07 μM) **MAO-B** (IC_50_ = 17.0 μM) **IL-1β reduction** (41.1%) **Antioxidant activity**
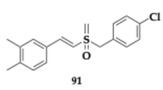	**4d** [98]	Styryl sulfone hybrid	**NO inhibition** (94.0%) **p38 MAPK** **NF-κB** **Nrf2**

**Table 17:** Potential multitargeted drugs for PD against MAO-B and other pathological targets.

Molecule	Molecule Name	Chemical Class	Primary Targets
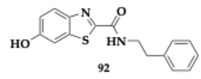	**30** [76]	6-hydroxybenzothiazol-2-carboxamide	**MAO-B** (IC_50_ = 0.041 μM) **tau** (EC_50_ = 10.5 μM) **α-syn** (IC_50_ = 9.09 μM)
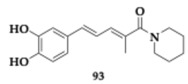	**8** [99]	Caffeic acid derivative	**COMT** (IC_50_ = 1.33 μM) **MAO-B** (IC_50_ = 4.27 μM)
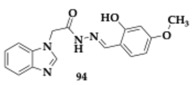	**7** [92]	Benzimidazole hybrid	**MAO-B inhibition**
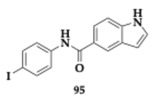	**8a** [100]	Indole derivative	**MAO-B** (IC_50_ = 0.02 μM)
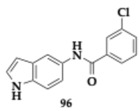	**3n** [101,102]	Melatonin hybrid	**MAO-B** (IC_50_ = 1.41 μM)

**Table 18:** Potential multitargeted drugs for PD against cholinesterases, MAOs, and other pathological elements.

Molecule	Molecule Name	Chemical Class	Primary Targets
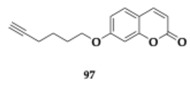	**9** [102]	Coumarin derivative	**MAO-B** (IC_50_ = 0.007 μM) **CA inhibition** **AChE inhibition** **BuChE inhibition**
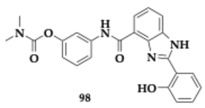	**5a** [103]	Rivastigmine-benzimidazole hybrid	**Aβ_42_ self- and Cu-induced aggregation inhibition** (45%) **AChE inhibition** **BuChE inhibition** **Metal chelation** **Antioxidant activity**
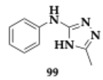	**4b** [104]	1,3,4-oxadiazole derivative	**AChE** (IC_50_ = 0.83 μM) **MAO-A** (IC_50_ = 1.44 μM) **MAO-B** (IC_50_ = 1.04 μM)
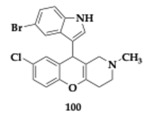	**3a** [105]	1H-chromeno[3,2-c]pyridine derivative	**AChE** (IC_50_ = 6.79 μM) **MAO-A** (IC_50_ = 8.42 μM) **MAO-B** (IC_50_ = 0.510 μM)
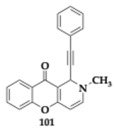	**6c** [105]	1H-chromeno[3,2-c]pyridine derivative	**AChE** **MAO-A** **MAO-B**
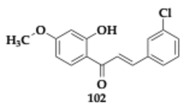	**2a** [106]	2′-hydroxychalcone derivative	**MAO-B** (IC_50_ = 0.111 μM) **Aβ_1–42_ aggregation inhibition** (75.7%) **GABA_A_ receptor** (Ki = 5.0 μM) **AChE inhibition**
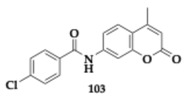	**10** [107]	7-amidocoumarin derivative	**MAO-A** **MAO-B** **AChE** **BuChE** **BACE-1**

**Table 19:** Potential multitargeted drugs for PD against α-syn and other pathological targets.

Molecule	Molecule Name	Chemical Class	Primary Targets
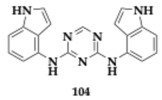	**10** [108]	Indole triazine derivative	**α-syn aggregation inhibition** (FI = 13.5%), **tau aggregation inhibition** **hyperphosphorylated tau oligomerization inhibition**
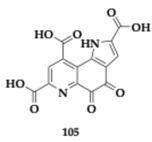	**PQQ** [109]	Pyrroloquinoline quinone	**Antioxidant activity** **α-syn Cu-induced aggregation inhibition**
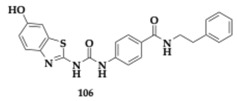	**24b** [110]	Hydroxybenzothiazole hybrid	**Dyrk1A inhibition** **α-syn aggregation inhibition** **tau aggregation inhibition**

**Table 20:** Potential multitargeted drugs for PD presenting agonism of dopamine receptor and antagonism of adenosine receptor.

Molecule	Molecule Name	Chemical Class	Primary Targets
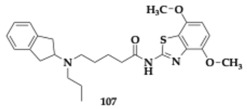	**30** [111]	Benzothiazole derivative	**A_2A_R** (Ki = 0.16 μΜ) **D_2_R** (Ki = 0.37 μΜ)

**Table 21:** Potential multitargeted drugs for PD presenting agonism of histamine receptor and antagonism of adenosine receptor.

Molecule	Molecule Name	Chemical Class	Primary Targets
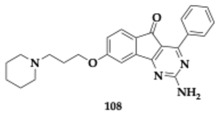	**12** [112]	Benzimidazolone derivative	**A_1_R** **A_2A_R** **H_3_R**

**Table 22:** Potential multitargeted drugs for HD.

Molecule	Molecule Name	Chemical Class	Primary Targets
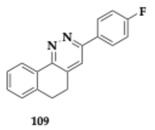	**3d** [113]	3-aryl-5,6-dihydrobenzo[h]cinnoline derivative	**AChE** **BuChE** **MAO-A** **MAO-B** **LIMK-2**
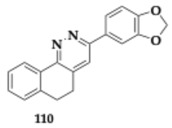	**3h** [113]	3-aryl-5,6-dihydrobenzo[h]cinnoline derivative	**AChE** **BuChE** **MAO-A** **MAO-B** **DHODH** **NMDAR** **JNKs**
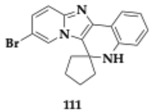	**5p** [114]	Quinoline hybrid	**AChE** **BuChE** **CB2 agonism**
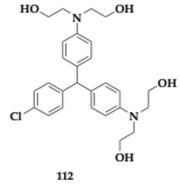	**LM22B** [115]	Triarylmethane derivative	**TrkB** **TrkC** **AKT**
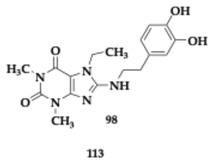	**11b** [116]	Xanthine derivative	**MAO-B** (IC50 = 47.9 nM) **A_2A_R** (Ki = 0.672 μM)
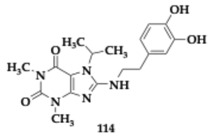	**11d** [116]	Xanthine derivative	**PDE4B1** (IC50 = 2.44 μM) **PDE10A** (IC50 = 2.30 μM)

## Data Availability

No new data were created in this study. Literature’s data were analyzed. Sources for the data are given through the references session.

## Abbreviations

The following abbreviations are used in this manuscript:

AD	Alzheimer’s Disease
HD	Huntington’s Disease
PD	Parkinson’s diseases
Aβ	Amyloid-beta peptides
AChEI	Acetylcholinesterase inhibitors
ACh	Acetylcholine
NMDA	*N*-methyl-D-aspartate
THA	Tacrine
ATP	Adenosine Triphosphate
ChEs	Cholinesterases
MAO	Monoamine oxidase
COMT	Catechol-*O*-methyltransferase
L-DOPA	L-3,4-Dihydroxyphenylalanine
MTDLs	Multitarget-directed ligands
ΒΒΒ	Blood–brain barrier
AchE	Acetylcholinesterase
BuChE	Butyrylcholinesterase
CAS	Catalytic active site
PAS	Peripheral anionic site
IC50	Inhibitory Concentration 50
EC50	Effective Concentration 50
SH-SY5Y	SK-N-SH human neuroblastoma cell lines
Neuro2A	Neuroblastoma-2A cell lines
PC12	Pheochromocytoma cell line 12
PAMPA	parallel artificial membrane permeability assay
ROS	Reactive Oxygen Species
NLRP30	Nucleotide-binding Oligomerization Domain like receptor 30
SAR	Structure–activity relationship
VEGF	Vascular endothelial growth factor
BV2	Murine microglial cell line
GSK-3β	Glycogen synthase kinase-3β
DYRK1A	Dual-specificity tyrosine phosphorylation-regulated kinase 1A
NFTs	Neurofibrillary tangles
HL-7702	Human liver 7702 cell lines
HepG2	Hepatocellular Gep 2 cells
THLE2	Telomerase-Immortalized Human Liver Epithelial Cells 2
CDK2	Cyclin-Dependent Kinase 2
HDAC	Histone Deacetylase
CAP	Catabolite activator protein
TNF-α	Tumor Necrosis Factor Alpha
IFN-γ	Interferon Gamma
A1	Astrocytes
H3R	Antagonism histamine H3 receptor
ADME	Absorption, Distribution, Metabolism, and Excretion
Ki	Inhibition constant
hCMEC/D3	Human cerebral microvascular endothelial cells
hPepT1-MDCK	Human Peptide Transporter 1 Madin-Darby Canine Kidney cells
FAAH	Fatty-acid amide hydrolase 1
LOX	Lipoxygenase
COX2	Cyclooxygenase-2
BACE-1	Beta-site Amyloid precursor protein Cleaving Enzyme-1
APP	Amyloid Precursor Protein
NF-κΒ	Nuclear Factor kappa-light-chain-enhancer of activated B cells
IL	Interleukin
CHL	Chinese Hamster Lung Cell Assay
CA	Carbonic anhydrase
FDA	Food and Drug Administration
Caco-2	Human colon carcinoma cell line
Nrf2	Nuclear factor erythroid 2-related factor 2
CatS	Cathepsin S
Keap1	Kelch-like ECH-associated protein 1
ARE	Antioxidant Response Element
AREc32	Antioxidant Response Element cell line 32
CD	Concentration
NOX2	NADPH oxidase isoform 2
NADPH	Nicotinamide Adenine Dinucleotide Phosphate
LRRK2	Leucine-rich repeat kinase 2
N27-A	Mouse midbrain dopaminergic neuronal cell lines
CYP3A4	Cytochrome P450 3A4
Δψm	Mitochondrial Membrane Potential
6-OHDA	6-hydroxydopamine
CREB	cAMP Response Element-Binding Protein
PGC-1α	Peroxisome Proliferator-Activated Receptor Gamma Coactivator 1-alpha
NRF-1	Nuclear Respiratory Factor 1
TFAM	Transcription Factor A Mitochondria
PKA	Protein Kinase A
Akt	Protein Kinase B
MAPK	Mitogen-activated protein kinase
HT-22	Mouse hippocampal cell lines
NO	Nitric Oxide
LPS	Lipopolysaccharide
MCF-7	Michigan Cancer Foundation breast cancer cell lines
HCT116	human colon cancer cell lines 116
SK-OV-3	Sankyo Ovarian 3 human ovarian adenocarcinoma cell lines
GABAA	γ-aminobutyric acid A
α-syn	Alpha-synuclein
FI	Fluorescence Intensity
A2AR	Adenosine A2A receptor
D2R	Dopamine receptor
LIMK-2	LIM-domain-containing protein kinase 2
DHODH	Dihydroorotate dehydrogenase
JNKs	c-Jun N-terminal kinases
NMDAR	*N*-Methyl-D-Aspartate Receptor
CB2	Cannabinoid receptor 2
TrkB	Tropomyosin receptor-kinase B
TrkC	Tropomyosin receptor-kinase C
LM22B-10	Microglial cell lines
A2AAR	Antagonism of A2A adenosine receptor
PDE	Phosphodiesterase
